# Spatio-temporal disparities of *Clonorchis* *sinensis* infection in animal hosts in China: a systematic review and meta-analysis

**DOI:** 10.1186/s40249-023-01146-4

**Published:** 2023-10-17

**Authors:** Kai Liu, Jing Tan, Lu Xiao, Rui-Tai Pan, Xiao-Yan Yao, Fu-Yan Shi, Shi‐Zhu Li, Lan‐Hua Li

**Affiliations:** 1 School of Public Health, Weifang Medical University, Weifang, 261053 China; 2 National Institute of Parasitic Diseases, Chinese Center for Disease Control and Prevention, Shanghai, 200025 China; 3 Key Laboratory of Parasite and Vector Biology, Ministry of Health, WHO Collaborating Center for Malaria, Schistosomiasis and Filariasis, Shanghai, 200025 China

**Keywords:** *Clonorchissinensis*, China, Prevalence, Spatio-temporal distribution, Biogeographical characteristics, Animal host, Meta-analysis

## Abstract

**Background:**

*Clonorchis* *sinensis*, one of the most important food-borne zoonotic trematodes, remains prevalent in China. Understanding its infection status in animals is crucial for controlling human clonorchiasis. Here we conducted a systematic review and meta-analysis to focus on the spatio-temporal disparities of *C.* *sinensis* infection in animals in China.

**Methods:**

Data on *C.* *sinensis* prevalence in snails, the second intermediate hosts, or animal reservoirs in China were extracted from electronic databases including PubMed, Embase, Web of Science, Chinese Wanfang database, CNKI, VIP, and China Biomedical Literature database. A random-effects meta-analysis model was utilized to estimate the pooled prevalence in each of the above animal hosts. Subgroup analysis and multivariable meta-regression were performed to explore potential sources of heterogeneity across studies and compare the temporal disparity of infection rates between high and low epidemic areas. Scatter plots were used to depict the biogeographical characteristics of regions reporting *C.* *sinensis* infection in animals.

**Results:**

The overall pooled prevalence of *C.* *sinensis* was 0.9% (95% *CI*: 0.6–1.2%) in snails, 14.2% (12.7–15.7%) in the second intermediate host, and 14.3% (11.4–17.6%) in animal reservoirs. Prevalence in low epidemic areas (with human prevalence < 1%) decreased from 0.6% (0.2–1.2%) before 1990 to 0.0% (0.0–3.6%) after 2010 in snails (*P* = 0.0499), from 20.3% (15.6–25.3%) to 8.8% (5.6–12.6%) in the second intermediate hosts (*P* = 0.0002), and from 18.3% (12.7–24.7%) to 4.7% (1.0–10.4%) in animal reservoirs. However, no similar decrease in prevalence was observed in high epidemic areas (with human prevalence ≥ 1.0%). *C.* *sinensis* infections were predominantly reported in areas with altitudes below 2346 m and annual cumulative precipitation above 345 mm and were mostly concentrated in eastern China.

**Conclusions:**

There are spatio-temporal disparities in the animal infections of *C.* *sinensis* in different areas of China. Animal infections are primarily concentrated in regions with low altitude and high precipitation. The results suggest that implementing One Health-based comprehensive measures targeting both humans and animals, especially in high epidemic areas, is essential for successful eradication of *C.* *sinensis* in China.

**Supplementary Information:**

The online version contains supplementary material available at 10.1186/s40249-023-01146-4.

## Background

Foodborne trematodes cause infection in humans via the consumption of contaminated food (raw fish, crustaceans, or vegetables), and pose a significant global health threat [1, 2]. Among these parasites, *Clonorchis* *sinensis*, also known as *Opisthorchis* *sinensis*, is responsible for clonorchiasis, a zoonotic parasitic disease that has been under-recognized but has affected approximately 35 million individuals worldwide [3, 4]. *C.* *sinensis* is endemic predominantly in China, the Republic of Korea, Japan, and other Asian countries or regions. In China alone, the number of *C.* *sinensis* infections has surpassed 15 million, making it a major public health concern [5]. *C.* *sinensis* is associated with various hepatobiliary diseases, including cholangitis, eosinophilic pneumonia, periductal hepatic fibrosis, and liver cirrhosis [6, 7]. Notably, *C.* *sinensis*, along with *Opisthorchis* *viverrini* and *Schistosoma* *haematobium*, has been classified as a Group I carcinogen by the International Agency for Research on Cancer (IARC) [8]. Numerous studies have demonstrated a clear link between *C.* *sinensis* infection and the development of cholangiocarcinoma [9], highlighting the urgent need for effective prevention measures and treatment strategies.

The life cycle of *C.* *sinensis* is characterized by a three-host system, with a snail serving as the first intermediate host and a freshwater fish typically acting as the second intermediate host, while the definitive host can vary from humans to other animal reservoirs [10]. The life cycle of *C.* *sinensis* involves various stages, including eggs that are excreted by the definitive host into the water. In freshwater snails, the eggs hatch into miracidium and develop into sporocysts, rediae, and cercariae. The cercariae then infect freshwater fish, where they transform into metacercariae [11]. Humans become infected with *C.* *sinensis* by consuming raw or undercooked freshwater fish containing the metacercariae stage [12, 13]. Given its three-host nature, the infection status of *C.* *sinensis* in animal hosts is closely related to the transmission of human clonorchiasis. Therefore, comprehending the level of infection in animals is essential for controlling human clonorchiasis.

Three national epidemiological surveys on major human parasitic diseases were conducted in China, spanning three time periods: 1988–1992, 2001–2004, and 2014–2016. These surveys provided relatively representative estimates for the prevalence of important parasitic diseases among Chinese residents. The results revealed significant regional disparities in the control of human clonorchiasis within China, with a noteworthy decrease in human prevalence observed in certain areas (Additional file 9: Table S1) [14–16]. However, provincial-level administrative divisions (PLADs) such as Guangdong, Guangxi, Jilin, and Heilongjiang continue to experience high human infection rates exceeding 1.0% [17]. Previous studies have reported varying infection rates in different animal hosts across different regions of China [18]. However, our understanding of the infection status of *C.* *sinensis* in various animal hosts and the spatio-temporal trends in these hosts remains limited. Furthermore, there is a lack of comprehensive research exploring the environmental and geographical aspects of animal infections.

Therefore, the objective of this study was to conduct a comprehensive investigation into the spatio-temporal distribution and biogeographical patterns of *C.* *sinensis* infections in animal hosts across China. Additionally, we aimed to estimate the prevalence of the parasite in both the first and second intermediate hosts, as well as in animal reservoirs, through a systematic review and meta-analysis. Furthermore, we sought to analyze the heterogeneity among the included studies and identify the factors that contribute to this heterogeneity. The findings of this study will contribute to a better understanding of the infection status of *C.* *sinensis* in animal hosts and provide valuable insights for the control of human clonorchiasis.

## Methods

### Literature retrieval and selection

This systematic review adhered to the Preferred Reporting Items for Systematic Reviews and Meta-analyses (PRISMA) reporting guidelines and has been registered with PROSPERO under the identifier CRD42023432917.

A comprehensive search was conducted in various online electronic databases to identify relevant studies on the survey of *C.* *sinensis* infections in animal hosts in China. The search encompassed the period from the inception of the databases to October 31, 2022. Both English and Chinese search terms were used, including terms such as ‘*Clonorchis* *sinensis*’, ‘*Clonorchis* *sinenses*’, ‘Clonorchiasis’, ‘*Opisthorchis* *sinensis*’, or ‘*Opisthorchis* *sinenses*’. The electronic databases that were searched included PubMed, Embase, Web of Science, Chinese Wanfang database (CWFD, https://www.wanfangdata.com.cn/), Chinese National Knowledge Infrastructure database (CNKI, https://www.cnki.net/), Chongqing VIP Chinese Science (VIP, http://qikan.cqvip.com/), and China Biomedical Literature database (CBM, http://www.sinomed.ac.cn/). This comprehensive search approach aimed to identify a wide range of studies relevant to the topic under investigation.

After removing duplicate records, the titles and abstracts of the remaining studies were independently reviewed by two reviewers (KL and LX). In case of any disagreements, a third reviewer (JT) provided assistance to reach a consensus. Subsequently, the full-text articles of potentially eligible studies were assessed by the same reviewers. Additionally, we manually searched the reference lists of the included publications to identify any additional relevant studies that may have been missed in the electronic search. The inclusion criteria for the studies were as follows: (1) English or Chinese epidemiological studies, (2) reporting the infection rate of *C.* *sinensis* in the first or second intermediate hosts or definitive hosts, and (3) primary research articles. On the other hand, publications without appropriate infection rate information (e.g., the numerator and/or denominator for the infection rate were inappropriate), or with a sample size of less than 20 were excluded from the analysis [19].

### Data extraction and quality assessment

The following variables were extracted from the eligible studies: study title, first author, year of publication, year of investigation, season of investigation, study locations, classification of population infection level, animal host species, detecting method, sample size, and the number of positive samples. These extracted data were recorded and organized in Additional file 10: Table S2, Additional file 11: Table S3, Additional file 12: Table S4.

Population infection levels were classified into two groups based on the infection rates of each PLAD as reported in the third national parasitic survey [20]. The classification groups were as follows: PLADs with a population infection rate ≥ 1.0%, which included Guangdong, Guangxi, Jilin, and Heilongjiang (high epidemic areas); PLADs with a population infection rate < 1.0%, which comprised all other PLADs except the aforementioned four PLADs (low epidemic areas). For the season of investigation, spring includes March to May, summer includes June to August, autumn includes September to November, and winter includes December to February.

After summarizing all the data, two authors (KL and JT) separately assessed the risk of bias of all included studies using the Hoy Risk of Bias Tool [21, 22]. This tool provides ten items to access the risk of bias, each given a score of 0 or 1 for the absence or presence of bias. A summary score of 0–3 indicates a low risk of bias, 4–6 a moderate risk of bias, and 7–10 a high risk of bias [23].

### Statistical analysis

We employed a double-arcsine transformation on the infection rates. This transformation helps to normalize the data distribution and ensure the validity of subsequent analyses [24]. After the transformation, we calculated the pooled infection rates and their corresponding 95% confidence intervals (*CI*s) for the animal hosts. To assess the heterogeneity among studies, we conducted Q-test and *I*^2^-value analyses. A lower *I*^2^-value suggests low heterogeneity, while moderate and high heterogeneity are indicated by *I*^2^-values between 25% and 50%, and greater than 50%, respectively [25]. In cases where the *P-*value from the Q-test was less than 0.1 and the *I*^2^-value was 50% or greater, indicating substantial heterogeneity, we utilized a random-effects model to combine effect sizes [26, 27]. We finally used the random-effects model to estimate the pooled prevalence in this study, taking into consideration the heterogeneity observed in the data. Additionally, we conducted subgroup and meta-regression analyses to explore potential sources of heterogeneity and assess the impact of various moderators on the infection rates. The regression model heterogeneity (QM) and residual error heterogeneity (QE) statistics were used to interpret the results of subgroup and meta-regression analyses [28]. The significance of unexplained residual heterogeneity was assessed using the QE statistic and its corresponding *P*-value, while the significance of the moderators was determined using the QM statistic and its *P*-value [29].

Funnel plots were used to evaluate potential publication bias, and Egger's test was performed to assess funnel plot asymmetry [30, 31]. To examine the robustness of the pooled prevalence estimates, sensitivity analyses were conducted. Outlier analyses were performed using Baujat plots. Studies located in the top right quadrant of the Baujat plot, or with studentized residuals exceeding 2 in absolute value, were considered potential outliers [32, 33]. We then assessed the impact of removing these identified outliers on the overall pooled prevalence estimates and compared the results to the main findings. Furthermore, we conducted sensitivity analyses to investigate the influence of studies with smaller sample sizes. Specifically, we examined whether excluding data points with the lowest quintile of sample sizes would yield similar findings to the main results. Finally, we examined if meta-analyses showed similar findings with the main results after excluding studies at moderate or high risk of bias. By conducting these sensitivity analyses, we aimed to assess the robustness and reliability of the main results, taking into account potential outliers, the influence of studies with smaller sample sizes, and the quality of publications.

Packages including ‘meta’ and ‘metafor’ in software R 4.0.5 (Lucent Technologies, Jasmine Mountain, USA) were used to conduct the meta-analysis. These packages are specifically designed for conducting meta-analyses and provide a range of functions and methods for data analysis and synthesis. A *P*-value less than 0.05 was considered to be statistically significant.

### Data collection on environmental factors and visualization of the spatio-temporal distribution and biogeographical characteristics

To gather geographical data for the survey sites, we utilized Baidu Maps to determine the latitude and longitude coordinates of each location. For climate data, we obtained information on the annual mean temperatures and annual cumulative precipitation from the WorldClim database. This database provides data at a resolution of 2.5 arc minutes and can be accessed at http://www.worldclim.org [34]. Altitude data was derived from the SRTMDEM data of the Geospatial Data Cloud, which offers data at a resolution of 90 m [35]. This data was obtained from http://www.gscloud.cn/.

To visualize the spatio-temporal distribution of *C.* *sinensis* infection in animal hosts, we georeferenced the infection rates of various animal hosts and plotted them on an epidemic map of China using software ArcGIS 10.7 (Environment System Research Institute, Redlands, USA). This allowed us to create a visual representation of the distribution across different regions. Additionally, to depict the biogeographical characteristics of regions reporting *C.* *sinensis* infection in animals, we utilized R 4.0.5 software to create a scatter plot. The scatter plot helps illustrate the relationships and patterns between infections and geographical factors.

## Results

### Literature selection and quality assessment

**Fig. 1:**
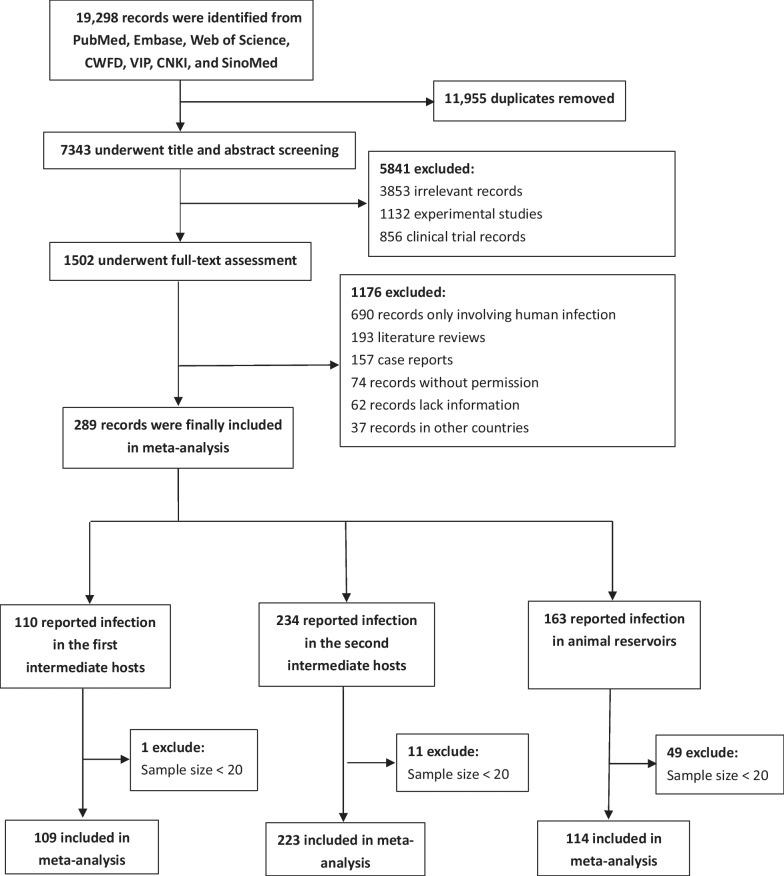
Flow diagram of study selection. Of all the articles included in the study, 38 included freshwater fish and freshwater snail infections, 12 included freshwater fish and reservoir hosts infections, 9 included freshwater snail and reservoir hosts infections, and 41 included freshwater fish, freshwater snail, and reservoir hosts infections

A total of 19,298 publications were identified through the online database search. After removing duplicates, 7343 were excluded based on title and abstract screening. Following full-text assessment, 289 publications were found to meet the inclusion criteria and were included in subsequent analyses. Of the included publications, 109 reported infections in freshwater snails, 223 in the second intermediate hosts, and 114 in animal definitive hosts (Fig. 1).

For the risk of bias assessment, risk of bias scores ranged from 2–5 (low to moderate biases). To be specific, 80 out of 109 publications for snails, 157 out of 223 for the second intermediate host, and 103 out of 114 for animal reservoirs were rated as low bias. The most common risk was lack of random selection of the sample or lack of reporting detecting method used to measure *C.* *sinensis* infection. The basic characteristics of the included articles, extracted data, and quality assessment results can be found in Additional file 10: Table S2, Additional file 11: Table S3, Additional file 12: Table S4.

### *C. sinensis* infection in the first intermediate hosts

A total of 109 articles covering 210 data points and 452,969 snail samples were finally included in the meta-analysis to estimate the pooled prevalence of *C.* *sinensis* infection in the first intermediate host. The prevalence of *C.* *sinensis* in snails ranged from 0.0 to 67.2% (Additional file 10: Table S2).

The pooled prevalence was estimated to be 0.9% (95% *CI*: 0.6–1.2%). The included studies exhibited high heterogeneity (*I*^2^ = 97.0%, *P* < 0.0001; see Table 1), and the corresponding forest plot is provided in Additional file 1: Fig. S1.

**Table 1:** Estimates of pooled prevalence and subgroup analysis of *Clonorchis* *sinensis* in first intermediate hosts

	All areas						
	No. of data points	Sample size	No. of positive	Pooled prevalence, % (95% *CI*)	*I*^*2*^, % (95% *CI*)	*R*^*2*^, % (QM *P* value)	QE *P* value
*All first intermediate hosts*	210	4,52,969	4528	0.9 (0.6–1.2)	97.0 (96.8–97.2)		
Taxonomic class						1.8 (0.1024)	< 0.0001
* Parafossarulus* *striatulus*	85	2,20,304	2592	1.1 (0.6–1.6)	97.4		
* Parafossarulus* *sinensis*	6	1825	50	3.5 (0.8–7.6)	93.4		
* Parafossarulus* *anomalospiralis*	2	269	19	10.1 (2.2–22.4)	98.1		
* Alocinma* *longicornis*	44	46,889	686	0.9 (0.3–1.7)	92.6		
* Bithynia* *fuchslana*	30	1,53,016	716	0.4 (0.0–1.2)	93.7		
* Bithynia* *robustu*	2	2514	50	2.0 (0.0–8.7)	0.0		
* Bithynia* *misella*	1	1068	2	0.2 (0.0–6.7)	Ne		
* Semisulcospira* *cancellata*	8	1544	21	0.1 (0.0–1.9)	78.5		
* Cipangopaludina* *chinensis*	8	4050	0	0.0 (0.0–1.0)	0.0		
* Lymnaea* sp.	3	743	0	0.0 (0.0–2.3)	0.0		
* Tricula* sp.	1	7743	0	0.0 (0.0–4.7)	Ne		
* Melanoides* *tuberculata*	1	71	0	0.0 (0.0–8.2)	Ne		
Unspecified	19	12,933	392	1.2 (0.3–2.7)	98.2		
Investigation period						1.5 (0.1421)	< 0.0001
Before 1990	106	2,85,066	2566	0.7 (0.4–1.1)	97.2		
1990–1999	37	95,008	1424	1.7 (0.9–2.8)	98.4		
2000–2009	61	70,191	530	0.9 (0.4–1.6)	91.3		
After 2010	6	2704	8	0.1 (0.0–1.9)	59.5		
Season of investigating						0.0 (0.3415)	< 0.0001
Spring	12	7655	49	0.6 (0.0–2.2)	93.0		
Summer	17	8191	33	0.2 (0.0–1.2)	79.9		
Autumn	21	10,904	82	0.7 (0.0–1.9)	90.3		
Winter	4	852	32	3.2 (0.2–8.9)	96.5		
Unspecified	156	4,25,367	4332	1.0 (0.6–1.4)	97.6		
*P.* *striatulus*	85	2,20,304	2592	1.1 (0.7–1.5)	97.4 (97.1–97.7)		
Investigation period						0.0 (0.5273)	< 0.0001
Before 1990	49	1,35,542	1921	1.0 (0.5–1.7)	98.0		
1990–1999	16	38,383	345	1.1 (0.3–2.4)	95.1		
2000–2009	16	44,263	321	1.5 (0.6–2.9)	95.9		
After 2010	4	2116	5	0.0 (0.0–1.8)	69.1		
Season of investigating						1.3 (0.2781)	< 0.0001
Spring	3	1304	5	0.5 (0.0–3.8)	88.7		
Summer	7	5220	25	0.6 (0.0–2.3)	91.8		
Autumn	10	5904	37	0.5 (0.0–1.9)	78.7		
Winter	2	405	30	6.0 (1.1–14.1)	98.0		
Unspecified	63	2,07,471	2495	1.2 (0.7–1.7)	97.9		

	Areas with population infection rate ≥ 1.0%						
	No. of data points	Sample size	No. of positive	Pooled prevalence, % (95% *CI*)	*I*^*2*^, % (95% *CI*)	*R*^*2*^, % (QM *P* value)	QE *P* value
*All first intermediate hosts*	102	1,45,762	1591	0.9 (0.5–1.3)	94.3 (93.7; 95.1)		
Taxonomic class						7.7 (0.0658)	< 0.0001
* Parafossarulus* *striatulus*	35	79,468	643	1.0 (0.5–1.7)	95.7		
* Parafossarulus* *sinensis*	4	1050	38	4.9 (1.7–9.5)	91.9		
* Parafossarulus* *anomalospiralis*							
* Alocinma* *longicornis*	24	25,609	497	1.1 (0.5–2.1)	92.3		
* Bithynia* *fuchslana*	13	24,351	327	1.2 (0.3–2.7)	79.6		
* Bithynia* *robustu*	2	2514	50	2.0 (0.0–6.8)	0.0		
* Bithynia* *misella*	1	1068	2	0.2 (0.0–4.4)	Ne		
* Semisulcospira* *cancellata*	5	1343	12	0.2 (0.0–2.2)	87.1		
* Cipangopaludina* *chinensis*	4	3520	0	0.0 (0.0–1.1)	0.0		
* Lymnaea* sp.	2	356	0	0.0 (0.0–2.3)	0.0		
* Tricula* sp.							
* Melanoides* *tuberculata*	1	71	0	0.0 (0.0–5.9)	Ne		
Unspecified	11	6412	13	0.1 (0.0–0.9)	72.1		
Investigation period						0.0 (0.8283)	< 0.0001
Before 1990	45	44,652	595	0.9 (0.4–1.5)	95.2		
1990–1999	16	59,764	729	0.8 (0.2–1.9)	95.0		
2000–2009	37	40,042	259	1.0 (0.5–1.8)	91.3		
After 2010	4	1304	8	0.2 (0.0–2.5)	0.0		
Season of investigating						0.0 (0.4813)	< 0.0001
Spring	4	2292	9	0.9 (0.0–4.1)	90.7		
Summer	11	3464	21	0.1 (0.0–1.1)	68.5		
Autumn	12	4386	48	1.0 (0.1–2.5)	93.0		
Winter	2	658	1	0.1 (0.0–2.9)	43.2		
Unspecified	73	1,34,962	1512	1.0 (0.6–1.5)	95.2		
*P.* *striatulus*	35	79,468	643	1.0 (0.5–1.7)	95.7 (94.8–96.5)		
Investigation period						0.0 (0.8437)	< 0.0001
Before 1990	18	29,426	356	1.2 (0.0–2.3)	96.4		
1990–1999	6	20,307	136	0.5 (0.0–2.2)	94.4		
2000–2009	9	29,019	146	1.3 (0.0–3.0)	95.4		
After 2010	2	716	5	0.3 (0.0–4.4)	0.0		
Season of investigating						0.0 (0.7911)	< 0.0001
Spring	1	109	5	4.6 (0.0–16.4)	Ne		
Summer	2	843	13	1.1 (0.0–5.4)	96.2		
Autumn	4	1548	10	0.4 (0.0–2.8)	45.0		
Winter	1	238	1	0.4 (0.0–6.5)	Ne		
Unspecified	27	76,730	614	1.1 (0.0–1.9)	96.6		

	Areas with population infection rate < 1.0%						
	No. of data points	Sample size	No. of positive	Pooled prevalence, % (95% *CI*)	*I*^*2*^, % (95% *CI*)	*R*^*2*^, % (QM *P* value)	QE *P* value
*All first intermediate hosts*	108	3,07,207	2937	0.9 (0.4–1.4)	97.9 (97.7–98.1)		
Taxonomic class						6.4 (0.0456)	< 0.0001
* Parafossarulus* *striatulus*	50	1,39,994	1949	1.1 (0.5–2.0)	97.9		
* Parafossarulus* *sinensis*	2	775	12	1.2 (0.0–8.0)	96.2		
* Parafossarulus* *anomalospiralis*	2	269	19	10.6 (1.8–24.6)	98.1		
* Alocinma* *longicornis*	20	21,280	189	0.6 (0.0–1.8)	89.5		
* Bithynia* *fuchslana*	17	1,28,665	389	0.1 (0.0–1.0)	84.0		
* Bithynia* *robustu*							
* Bithynia* *misella*							
* Semisulcospira* *cancellata*	3	201	0	0.0 (0.0–4.0)	0.0		
* Cipangopaludina* *chinensis*	4	530	0	0.0 (0.0–2.7)	0.0		
* Lymnaea* sp.	1	387	0	0.0 (0.0–6.9)	Ne		
* Tricula* sp.	1	7743	0	0.0 (0.0–5.8)	Ne		
* Melanoides* *tuberculata*							
Unspecified	8	1136	33	3.9 (1.2–8.0)	99.6		
Investigation period						5.5 (0.0499)	< 0.0001
Before 1990	61	2,40,414	1971	0.6 (0.2–1.2)	97.9		
1990–1999	21	35,244	695	2.7 (1.1–4.7)	99.0		
2000–2009	24	30,149	271	0.6 (0.0–1.8)	90.9		
After 2010	2	1400	0	0.0 (0.0–3.6)	0.0		
Season of investigating						4.1 (0.0724)	< 0.0001
Spring	8	5363	40	0.5 (0.0–2.7)	94.3		
Summer	6	4727	12	0.4 (0.0–2.8)	86.5		
Autumn	9	6518	34	0.4 (0.0–2.4)	83.6		
Winter	2	194	31	13.0 (2.5–29.0)	37.7		
Unspecified	83	2,90,405	2820	0.9 (0.4–1.5)	98.3		
*P.* *striatulus*	50	1,40,836	1949	1.1 (0.6–1.8)	97.9 (97.6–98.1)		
Investigation period						0.7 (0.3439)	< 0.0001
Before 1990	31	1,06,116	1565	0.9 (0.0–1.8)	98.4		
1990–1999	10	18,076	209	1.6 (0.0–3.8)	95.5		
2000–2009	7	15,244	175	1.9 (0.0–4.7)	95.9		
After 2010	2	1400	0	0.0 (0.0–2.4)	0.0		
Season of investigating						16.7 (0.0107)	< 0.0001
Spring	2	1195	0	0.0 (0.0–2.4)	0.0		
Summer	5	4377	12	0.5 (0.0–2.4)	89.2		
Autumn	6	4356	27	0.6 (0.0–2.5)	86.1		
Winter	1	167	29	17.4 (5.4–34.0)	Ne		
Unspecified	36	1,30,741	1881	1.2 (0.0–2.0)	98.3		

*CI* confidence interval, *QM* the regression model heterogeneity, *QE* the residual error heterogeneity

**Fig. 2:**
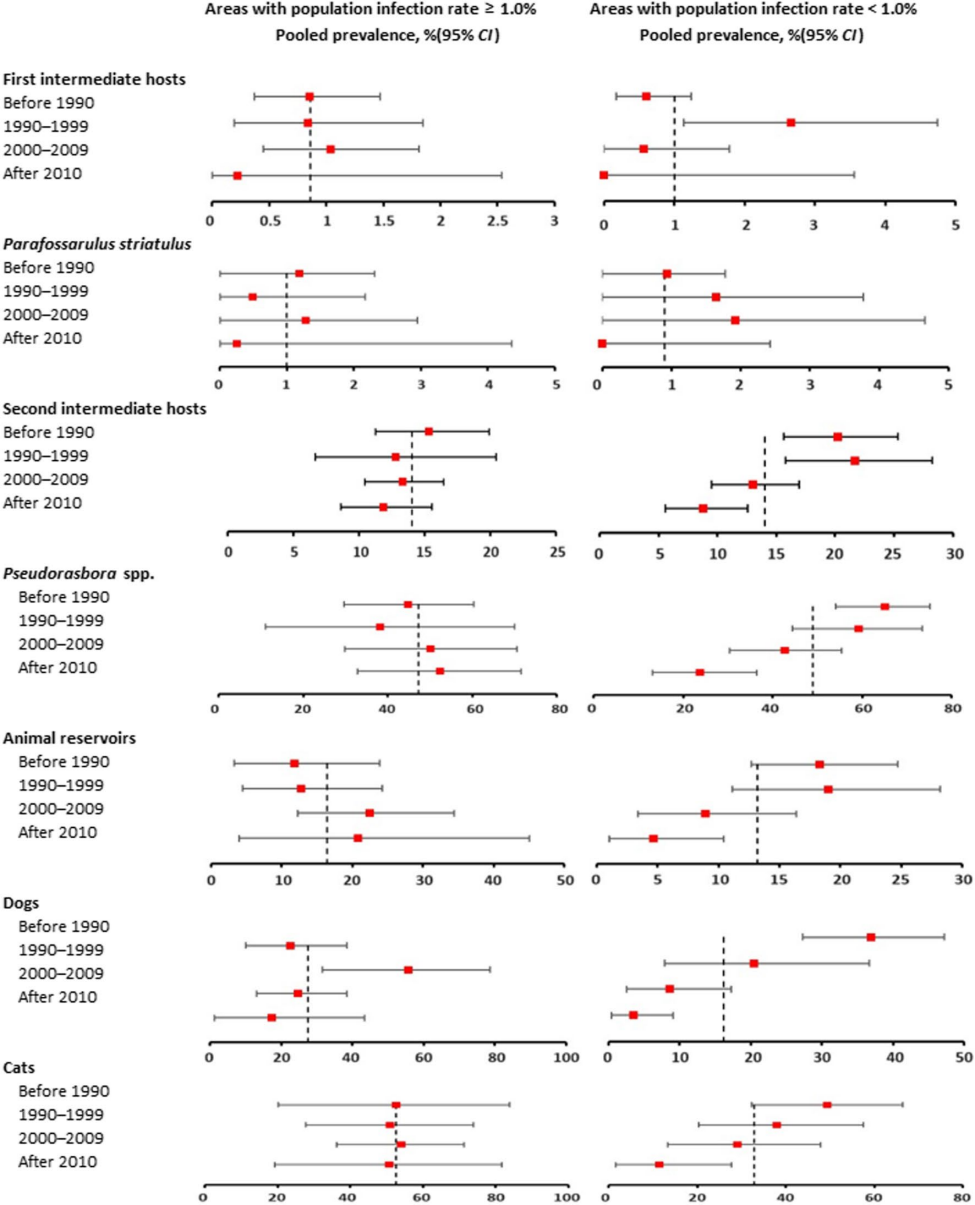
Temporal disparities of *Clonorchis* *sinensis* infection in animal hosts according to human infection level

The snail species that were most commonly reported to be infected with *C.* *sinensis* were *Parafossarulus* *striatulus* (pooled prevalence 1.1%, 95% *CI*: 0.6–1.6%), followed by *Alocinma* *longicornis* (0.9%, 95% *CI*: 0.3–1.7%) and *Bithynia* *fuchslana* (0.4%, 95% *CI*: 0.1–1.2%). Other potential vectors included *P.* *sinensis*, *P.* *anomalospiralis*, *B.* *robust*, *B.* *misella*, and *Semisulcospira* *cancellata*. When analyzed according to infection level in humans, infection rates were similar in high epidemic areas (PLADs with human prevalence ≥ 1.0%) and low epidemic areas (PLADs with human prevalence < 1.0%). The overall pooled prevalence of all snails was 0.9% (95% *CI*: 0.5–1.3%) in high epidemic areas, and 0.9% (95% *CI*: 0.4–1.4%) in low epidemic areas (see Table 1, Fig. 2).

When stratified by year of investigation, the pooled prevalence (95% *CI*) of all snails changed from 0.7% (0.4–1.1%) before 1990 to 0.1% (0.0–1.9%) after 2010 (*R*^*2*^ = 1.5, *P* = 0.1421). It changed from 0.9% (0.4–1.5%) before 1990 to 0.2% (0.0–2.5%) after 2010 (*R*^2^ = 0.0, *P* = 0.8283) in high epidemic areas, and from 0.6% (0.2–1.2%) to 0.0% (0.0–3.6%) in low epidemic areas (*R*^2^ = 5.5, *P* = 0.0499). When stratified by season of investigation, infection rates among different subgroups were similar.

Infection in *P.* *striatulus*, the most common vector, was further analyzed separately. The pooled prevalence (95% *CI*) in *P.* *striatulus* was 1.0% (0.5–1.7%) in high epidemic areas, and 1.1% (0.6–1.8%) in low epidemic areas (see Table 1, Fig. 2). When stratified by year of investigation, the overall pooled prevalence (95% *CI*) in *P.* *striatulus* changed from 1.0% (0.5–1.7%) to 0.0% (0.0–1.8%; *R*^2^ = 1.5, *P* = 0.1421). To be specific, it changed from 1.2% (0.0–2.3%) to 0.3% (0.0–4.4%) in high epidemic areas (*R*^2^ = 0.0, *P* = 0.8437), and from 0.9% (0.0–1.8%) to 0.0% (0.0–2.4%) in low epidemic areas (*R*^2^ = 0.7, *P* = 0.3439). Similar to the results of subgroup analysis, significant decrease in prevalence over time was not revealed in the meta-regression model (see Additional file 13: Table S5). However, it needs to be cautious in explaining the results because only 4 data points were included in the analysis for period after 2010.

### *C. sinensis* infection in the second intermediate hosts

A total of 223 articles covering 927 data points and 162,568 samples were included in the meta-analysis to estimate the pooled prevalence of *C.* *sinensis* infection in the second intermediate host.

The prevalence of *C.* *sinensis* infection in the second intermediate host varied widely, ranging from 0.0% to 100.0% (see Additional file 11: Table S3). The pooled prevalence was estimated to be 14.2% (95% *CI*: 12.7–15.7%), and the *I*^*2*^ value indicated high heterogeneity (98.6%, *P* < 0.0001, see Table 2; the forest plot is shown in Additional file 2: Fig. S2).

**Table 2:** Estimates of pooled prevalence and subgroup analysis of *Clonorchis* *sinensis* in second intermediate hosts

	All areas						
	No. of data points	Sample size	No. of positive	Pooled prevalence, % (95% *CI*)	*I*^*2*^, % (95% *CI*)	*R*^*2*^, % (QM *P* value)	QE *P* value
All second intermediate hosts	927	1,62,568	36,050	14.2 (12.7–15.7)	98.6 (98.6–98.7)		
*Taxonomic* *class*						35.5 (< 0.0001)	< 0.0001
Cyprinidae: Gobioninae							
*Pseudorasbora*	144	46,390	20,358	48.5 (44.2–52.7)	99.1		
*Abbottina*	40	5926	1800	28.7 (21.6–36.4)	99.0		
*Saurogobio*	12	2396	430	38.9 (24.8–54.0)	98.0		
*Hemibarbus*	3	195	14	4.7 (0.0–25.8)	81.0		
*Gnathopogon*	1	109	9	8.3 (0.0–51.7)	Ne		
*Paraleucogobio*	1	85	5	5.9 (0.0–47.7)	Ne		
*Mesogobio*	1	119	9	7.6 (0.0–50.4)	Ne		
Cyprinidae: Cyprininae							
*Carassius*	92	15,014	1267	7.2 (4.6–10.4)	95.5		
*Cyprinus*	55	4736	380	6.1 (2.9–10.2)	83.4		
Cyprinidae: Leuciscinae							
*Ctenopharyngodon*	70	12,718	3984	15.2 (10.9–20.1)	98.6		
*Squaliobarbus*	2	859	97	9.1 (0.0–38.2)	44.4		
*Pseudaspius*	1	145	108	74.5 (26.0–100.0)	Ne		
*Phoxinus*	5	608	80	19.2 (4.2–40.8)	93.1		
*Leuciscus*	1	27	4	14.8 (0.0–65.9)	Ne		
Cyprinidae: Culterinae							
*Hemiculter*	55	9576	1048	10.9 (6.9–15.7)	95.4		
*Parabramis*	26	1255	143	5.2 (1.2–11.3)	89.4		
*Erythroculter*	5	1200	139	16.7 (3.0–37.5)	92.8		
*Anabarilius*	2	191	89	46.8 (14.5–80.7)	93.7		
*Pseudolaubuca*	8	1502	153	11.8 (2.2–26.6)	81.9		
*Culter*	4	217	87	18.8 (2.5–43.7)	94.5		
*Megalobrama*	2	149	39	19.7 (0.5–54.0)	96.2		
*Pseudohemiculter*	1	2342	396	16.9 (0.0–63.3)	Ne		
Cyprinidae: Hypophthaemichthyinae							
*Aristichthys*	41	4821	766	11.6 (6.6–17.5)	90.7		
*Hypophthalmichthys*	31	1864	158	6.1 (2.0–11.9)	84.4		
Cyprinidae: Acheilognathinae							
*Rhodeus*	39	5381	880	14.7 (9.2–21.2)	97.9		
*Gobio*	2	41	7	11.6 (0.0–46.9)	91.6		
Cyprinidae: Labeoninae							
*Cirrhinus*	29	2748	496	9.8 (4.6–16.6)	94.4		
*Sinilabeo*	1	73	5	6.9 (0.0–49.9)	Ne		
*Ptychidio*	1	72	3	4.2 (0.0–44.3)	Ne		
Other fish in Cyprinidae							
*Xenocypris*	3	898	41	2.4 (0.0–21.6)	0.0		
*Distoechodon*	1	21	1	4.7 (0.0–51.5)	Ne		
*Opsariichthys*	4	346	45	8.6 (0.0–28.4)	85.4		
*Zacco*	1	732	130	17.8 (0.0–64.5)	Ne		
*Onychostoma*	1	93	7	7.5 (0.0–50.7)	Ne		
*Puntius*	1	152	5	3.3 (0.0–40.7)	Ne		
*Spinibarbus*	1	82	3	3.7 (0.0–42.9)	0.0		
Cypriniformes: Cobitidae							
*Misgurnus*	20	3667	138	3.9 (0.3–10.0)	96.3		
*Schistura*	1	153	44	28.8 (0.2–77.2)	Ne		
Perciformes: Cichlidae							
*Oreochromis*	20	2510	156	4.6 (0.7–11.1)	84.1		
*Cichla*	2	119	4	4.7 (0.0–32.4)	80.2		
Perciformes: Eleotridae							
*Perccottus*	8	865	261	40.5 (23.4–58.9)	98.6		
*Micropercops*	4	216	28	16.7 (1.6–40.9)	96.1		
*Bosttychus*	4	150	7	5.3 (0.0–24.2)	75.0		
*Odontobutis*	1	58	0	0.0 (0.0–29.4)	Ne		
Other fish in Perciformes							
*Channa*	9	459	23	4.7 (0.0–15.6)	80.9		
*Macropodus*	4	187	38	12.4 (0.4–34.8)	88.7		
*Lateolabrax*	5	219	3	0.7 (0.0–12.0)	0.0		
*Siniperca*	4	298	25	13.8 (0.6–36.8)	97.1		
*Epinephelus*	3	146	5	2.2 (0.0–20.5)	35.7		
*Rhinogobius*	8	1045	59	5.6 (0.0–17.7)	82.6		
*Pampus*	2	61	5	7.3 (0.0–38.4)	0.0		
*Mastacembelus*	1	33	33	100.0 (67.7–100.0)	Ne		
*Caranx*	1	31	1	3.2 (0.0–45.4)	Ne		
*Helostoma*	1	26	3	11.5 (0.0–61.6)	Ne		
Other fish except Perciformes and Cypriniformes							
*Pelteobagrus*	16	993	37	3.0 (0.0–9.9)	88.4		
*Silurus*	11	411	17	2.8 (0.0–11.5)	58.8		
*Clarias*	1	29	1	3.5 (0.0–46.4)	Ne		
*Monopterus*	10	1767	32	2.0 (0.0–9.8)	90.2		
*Salmo*	4	162	5	1.3 (0.0–15.4)	72.5		
*Brachymystax*	1	791	86	10.9 (0.0–54.9)	Ne		
*Salanx*	1	48	0	0.0 (0.0–30.4)	Ne		
*Oryzias*	3	95	8	7.0 (0.0–31.1)	30.2		
*Gambusia*	2	142	4	2.9 (0.0–26.8)	0.0		
*Sebastiscus*	1	39	5	12.8 (0.0–61.6)	Ne		
*Sardinella*	1	44	0	0.0 (0.0–30.7)	Ne		
Unspecified fish	53	15,501	1728	6.8 (3.5–10.8)	98.6		
Shellfish	38	9220	108	0.7 (0.0–3.3)	88.1		
Period of investigating						1.8 (0.0002)	< 0.0001
Before 1990	224	47,688	14,544	18.2 (14.9–21.6)	99.1		
1990–1999	114	19,024	4982	19.0 (14.5–24.0)	98.7		
2000–2009	337	56,251	10,831	13.2 (10.9–15.7)	98.2		
After 2010	252	39,605	5693	10.2 (7.8–12.8)	97.8		
Season of investigating						2.3 (< 0.0001)	< 0.0001
Spring	65	14,199	4775	29.2 (22.4–36.6)	98.9		
Summer	93	11,611	2029	13.9 (9.5–18.9)	98.0		
Autumn	115	14,358	3815	10.5 (7.0–14.6)	98.4		
Winter	13	1076	248	7.6 (0.6–20.0)	98.9		
Unspecified	641	1,21,324	25,183	13.7 (12.0–15.5)	98.7		
Detecting method						0.0 (0.6348)	< 0.0001
Direct compression	447	74,958	16,766	14.2 (12.1–16.5)	98.7		
Artificial digestion	237	34,458	6938	13.0 (10.3–16.0)	97.9		
Unspecified	243	53,152	12,346	15.1 (12.2–18.2)	99.0		
*Pseudorasbora* spp.	144	46,390	20,358	48.5 (42.8–54.3)	99.1 (99.0–99.1)		
Period of investigating						6.9 (0.0046)	< 0.0001
Before 1990	52	22,357	11,905	58.2 (49.0–67.2)	98.6		
1990–1999	23	5226	2474	55.6 (41.6–69.2)	98.5		
2000–2009	36	7360	2882	44.8 (33.8–56.0)	98.9		
After 2010	33	11,447	3097	32.7 (22.3–44.0)	99.2		
Season of investigating						3.6 (0.0562)	< 0.0001
Spring	12	1671	825	68.2 (48.7–85.0)	99.6		
Summer	19	3275	1280	38.7 (24.2–54.4)	98.5		
Autumn	20	5699	2941	46.6 (31.8–61.8)	98.8		
Winter	2	322	244	89.2 (47.0–1.0)	98.8		
Unspecified	91	35,423	15,068	47.3 (40.3–54.5)	99.1		
Detecting method						0.7 (0.2261)	< 0.0001
Direct compression	80	25,865	10,476	46.0 (38.3–53.7)	99.3		
Artificial digestion	20	3291	1540	42.3 (27.3–58.0)	96.9		
Unspecified	44	17,234	8342	55.9 (45.5–66.1)	98.6		

	Areas with population infection rate ≥ 1.0%						
	No. of data points	Sample size	No. of positive	Pooled prevalence, % (95% *CI*)	*I*^*2*^, % (95% *CI*)	*R*^*2*^, % (QM *P* value)	QE *P* value
All second intermediate hosts	425	69,593	13,703	13.3 (11.4–15.3)	97.8 (97.7–97.9)		
*Taxonomic* *class*						29.9 (< 0.0001)	< 0.0001
Cyprinidae: Gobioninae							
*Pseudorasbora*	43	8304	3668	47.3 (40.1–54.7)	97.8		
*Abbottina*	3	301	112	28.8 (8.2–55.6)	97.1		
*Saurogobio*	9	2126	334	35.2 (20.6–51.4)	98.2		
*Hemibarbus*	3	195	14	4.7 (0.0–23.9)	81.0		
*Gnathopogon*	1	109	9	8.3 (0.0–48.1)	Ne		
*Paraleucogobio*							
*Mesogobio*							
Cyprinidae: Cyprininae							
*Carassius*	37	5819	688	8.0 (4.1–12.9)	95.8		
*Cyprinus*	30	3510	299	8.1 (3.6–13.8)	80.8		
Cyprinidae: Leuciscinae							
*Ctenopharyngodon*	50	10,476	3572	18.6 (13.5–24.3)	98.7		
*Squaliobarbus*	2	859	97	9.1 (0.0–35.7)	44.4		
*Pseudaspius*	1	145	108	74.5 (29.3–99.9)	Ne		
*Phoxinus*	2	484	67	33.0 (6.8–66.6)	97.5		
*Leuciscus*							
Cyprinidae: Culterinae							
*Hemiculter*	19	2468	502	16.5 (9.1–25.5)	96.1		
*Parabramis*	17	738	115	6.7 (1.5–14.4)	91.7		
*Erythroculter*	2	1095	122	20.7 (1.6–52.1)	95.5		
*Anabarilius*							
*Pseudolaubuca*	4	1291	139	19.8 (4.1–42.5)	86.7		
*Culter*	1	37	11	29.7 (0.1–77.5)	Ne		
*Megalobrama*	1	57	3	5.3 (0.0–43.8)	Ne		
*Pseudohemiculter*	1	2342	396	16.9 (0.0–59.7)	Ne		
*Aristichthys*	32	4241	670	10.4 (5.5–16.4)	92.3		
*Hypophthalmichthys*	15	1101	109	8.0 (2.0–16.7)	83.3		
Cyprinidae: Acheilognathinae							
*Rhodeus*	13	1640	600	26.9 (15.9–39.6)	97.9		
*Gobio*	1	21	0	0 (0–32.29)	Ne		
Cyprinidae: Labeoninae							
*Cirrhinus*	26	2578	487	10.6 (5.3–17.4)	94.7		
*Sinilabeo*	1	73	5	6.9 (0.0–46.3)	Ne		
*Ptychidio*	1	72	3	4.2 (0.0–40.7)	Ne		
Other fish in Cyprinidae							
*Xenocypris*	2	873	41	4.5 (0.0–29.1)	0.0		
*Distoechodon*							
*Opsariichthys*	1	51	3	5.9 (0.0–45.4)	Ne		
*Zacco*	1	732	130	17.8 (0.0–61.0)	Ne		
*Onychostoma*	1	93	7	7.5 (0.0–47.1)	Ne		
*Puntius*							
*Spinibarbus*	1	82	3	3.7 (0.0–39.2)	Ne		
Cypriniformes: Cobitidae							
*Misgurnus*	8	1102	42	1.5 (0.0–9.1)	68.9		
*Schistura*	1	153	44	28.8 (0.7–73.9)	Ne		
Perciformes: Cichlidae							
*Oreochromis*	17	2367	151	5.1 (0.9–11.8)	86.1		
*Cichla*							
Perciformes: Eleotridae							
*Perccottus*	6	738	167	30.2 (14.1–49.3)	98.6		
*Micropercops*							
*Bosttychus*	1	23	2	8.7 (0–54.5)	Ne		
*Odontobutis*	1	58	0	0.0 (0.0–26.2)	Ne		
Other fish in Perciformes							
*Channa*	5	339	19	7.4 (0.0–23.0)	89.4		
*Macropodus*	1	75	27	36.0 (2.4–81.3)	Ne		
*Lateolabrax*	3	168	3	1.6 (0.0–17.2)	44.0		
*Siniperca*	4	298	25	13.6 (1.0–34.8)	97.1		
*Epinephelus*							
*Rhinogobius*	1	619	31	5.0 (0.0–40.3)	Ne		
*Pampus*	1	25	1	4.0 (0.0–45.0)	Ne		
*Mastacembelus*							
*Caranx*							
*Helostoma*							
*Pelteobagrus*	5	598	6	0.7 (0.0–10.0)	59.2		
*Silurus*	7	301	7	1.2 (0.0–9.8)	30.8		
*Clarias*	1	29	1	3.5 (0.0–42.8)	Ne		
*Monopterus*	4	461	32	9.9 (0.3–28.6)	84.6		
*Salmo*	2	99	5	3.8 (0.0–27.5)	89.2		
*Brachymystax*	1	791	86	10.9 (0.0–51.2)	Ne		
*Salanx*	1	48	0	0.0 (0.0–27.0)	Ne		
*Oryzias*							
*Gambusia*							
*Sebastiscus*							
*Sardinella*	1	44	0	0.0 (0.0–27.4)	Ne		
Unspecified fish	25	7636	710	3.4 (0.6–7.9)	97.0		
Shellfish	8	1708	30	1.7 (0.0–9.2)	92.4		
Period of investigating						0.0 (0.6575)	< 0.0001
Before 1990	95	10,354	2169	15.3 (11.2–19.9)	97.7		
1990–1999	32	6976	1642	12.8 (6.7–20.4)	98.9		
2000–2009	178	35,725	6539	13.3 (10.5–16.5)	97.7		
After 2010	120	16,538	3353	11.9 (8.6–15.5)	97.6		
Season of investigating						4.7 (< 0.0001)	< 0.0001
Spring	39	9732	3379	26.6 (19.1–34.9)	98.7		
Summer	37	4246	426	7.4 (3.1–13.1)	97.1		
Autumn	42	5657	1144	8.0 (3.7–13.6)	97.9		
Winter	3	164	1	0.1 (0.0–16.0)	0.0		
Unspecified	304	49,794	8753	13.6 (11.4–15.9)	97.3		
Detecting method						0.1 (0.2996)	< 0.0001
Direct compression	167	30,699	5597	14.3 (11.2–17.6)	98.1		
Artificial digestion	181	28,403	6017	11.7 (9.1–14.6)	97.7		
Unspecified	77	10,491	2089	15.3 (10.7–20.4)	97.5		
*Pseudorasbora* spp.	43	8304	3668	47.3 (37.7–57.1)	97.8 (97.4; 98.1)		
Period of investigating						0.0 (0.8666)	< 0.0001
Before 1990	18	2694	1097	44.8 (29.7–60.3)	97.1		
1990–1999	4	605	204	38.3 (11.1–70.1)	97.7		
2000–2009	10	1730	684	50.1 (29.8–70.5)	98.8		
After 2010	11	3275	1683	52.4 (32.9–71.5)	96.3		
Season of investigating						2.4 (0.2716)	< 0.0001
Spring	5	228	164	68.2 (39.5–91.2)	96.3		
Summer	7	729	236	31.1 (11.7–54.7)	95.3		
Autumn	5	1265	758	47.0 (20.5–74.5)	96.6		
Winter							
Unspecified	26	6082	2510	47.9 (35.8–60.2)	98.0		
Detecting method				0.0 (0.0–0.0)	0.0	6.5 (0.0795)	< 0.0001
Direct compression	21	4469	1926	51.3 (38.0–64.6)	98.3		
Artificial digestion	13	2748	1238	32.0 (17.1–48.9)	97.4		
Unspecified	9	1087	504	60.6 (39.8–79.7)	96.2		

	Areas with population infection rate < 1.0%						
	No. of data points	Sample size	No. of positive	Pooled prevalence, % (95% *CI*)	*I*^*2*^, % (95% *CI*)	*R*^*2*^, % (QM *P* value)	QE *P* value
All second intermediate hosts	502	92,975	22,347	14.9 (12.7–17.2)	99.0 (98.9–99.0)		
Taxonomic class						39.3 (< 0.0001)	< 0.0001
Cyprinidae: Gobioninae							
*Pseudorasbora*	101	38,086	16,690	49.0 (43.7–54.3)	99.3		
*Abbottina*	37	5625	1688	28.7 (20.9–37.1)	99.1		
*Saurogobio*	3	270	96	49.8 (20.1–79.6)	96.1		
*Hemibarbus*							
*Gnathopogon*							
*Paraleucogobio*	1	85	5	5.9 (0.0–50.2)	Ne		
*Mesogobio*	1	119	9	7.7 (0.0–52.9)	Ne		
Cyprinidae: Cyprininae							
*Carassius*	55	9195	579	6.8 (3.4–11.1)	94.7		
*Cyprinus*	25	1226	81	3.9 (0.4–9.8)	84.7		
Cyprinidae: Leuciscinae							
*Ctenopharyngodon*	20	2242	412	7.8 (2.2–15.9)	97.3		
*Squaliobarbus*							
*Pseudaspius*							
*Phoxinus*	3	124	13	11.1 (0.0–38.4)	87.4		
*Leuciscus*	1	27	4	14.8 (0.0–68.3)	Ne		
Cyprinidae: Culterinae							
*Hemiculter*	36	7108	546	8.4 (3.9–14.1)	92.7		
*Parabramis*	9	517	28	3.0 (0.0–13.1)	57.8		
*Erythroculter*	3	105	17	14.1 (0.0–42.8)	93.9		
*Anabarilius*	2	191	89	46.8 (13.3–82.1)	93.7		
*Pseudolaubuca*	4	211	14	5.6 (0.0–25.1)	73.3		
*Culter*	3	180	76	15.3 (0.0–44.9)	96.3		
*Megalobrama*	1	92	36	39.1 (1.6–87.9)	Ne		
*Pseudohemiculter*							
*Aristichthys*	9	580	96	16.3 (4.7–32.3)	64.4		
*Hypophthalmichthys*	16	763	49	4.6 (0.2–12.7)	84.4		
Cyprinidae: Acheilognathinae							
*Rhodeus*	26	3741	280	9.8 (4.2–17.2)	94.7		
*Gobio*	1	20	7	35.0 (0.0–89.0)	Ne		
Cyprinidae: Labeoninae							
*Cirrhinus*	3	170	9	4.2 (0.0–25.7)	51.9		
*Sinilabeo*							
*Ptychidio*							
Other fish in Cyprinidae							
*Xenocypris*	1	25	0	0.0 (0.0–36.6)	Ne		
*Distoechodon*	1	21	1	4.8 (0.0–54.0)	Ne		
*Opsariichthys*	3	295	42	9.6 (0.0–35.0)	89.1		
*Zacco*							
*Onychostoma*							
*Puntius*	1	152	5	3.3 (0.0–43.3)	Ne		
*Spinibarbus*							
Cypriniformes: Cobitidae							
*Misgurnus*	12	2565	96	6.0 (0.4–16.1)	97.6		
*Schistura*							
Perciformes: Cichlidae							
*Oreochromis*	3	143	5	2.2 (0.0–21.6)	33.8		
*Cichla*	2	119	4	4.8 (0.0–34.1)	80.2		
Perciformes: Eleotridae							
*Perccottus*	2	127	94	73.4 (35.1–98.4)	0.0		
*Micropercops*	4	216	28	16.7 (1.2–42.2)	96.1		
*Bosttychus*	3	127	5	4.4 (0.0–27.3)	80.2		
*Odontobutis*							
Other fish in Perciformes							
*Channa*	4	120	4	1.8 (0.0–18.6)	26.8		
*Macropodus*	3	112	11	6.3 (0.0–30.8)	69.0		
*Lateolabrax*	2	51	0	0.0 (0.0–21.2)	0.0		
*Siniperca*							
*Epinephelus*	3	146	5	2.2 (0.0–21.7)	35.7		
*Rhinogobius*	7	426	28	5.8 (0.0–19.9)	85.1		
*Pampus*	1	36	4	11.1 (0.0–61.9)	Ne		
*Mastacembelus*	1	33	33	100.0 (65.3–100.0)	Ne		
*Caranx*	1	31	1	3.2 (0.0–47.9)	Ne		
*Helostoma*	1	26	3	11.5 (0.0–64.1)	Ne		
*Pelteobagrus*	11	395	31	4.7 (0.0–15.1)	90.4		
*Silurus*	4	110	10	7.3 (0.0–29.3)	66.8		
*Clarias*							
*Monopterus*	6	1306	0	0.0 (0.0–6.7)	0.0		
*Salmo*	2	63	0	0.0 (0.0–20.3)	0.0		
*Brachymystax*							
*Salanx*							
*Oryzias*	3	95	8	7.0 (0.0–32.4)	30.2		
*Gambusia*	2	142	4	2.9 (0.0–28.4)	0.0		
*Sebastiscus*	1	39	5	12.8 (0.0–64.0)	Ne		
*Sardinella*							
Unspecified fish	28	7865	1018	10.5 (5.0–17.5)	99.1		
Shellfish	30	7512	78	0.5 (0.0–3.5)	86.6		
Period of investigating						3.5 (0.0002)	< 0.0001
Before 1990	129	37,334	12,375	20.3 (15.6–25.3)	99.4		
1990–1999	82	12,048	3340	21.7 (15.8–28.2)	98.6		
2000–2009	159	20,526	4292	13.0 (9.5–17.0)	98.6		
After 2010	132	23,067	2340	8.8 (5.6–12.6)	97.6		
Season of investigating						1.8 (0.0103)	< 0.0001
Spring	26	4467	1396	33.2 (21.3–46.2)	99.2		
Summer	56	7365	1603	19.2 (12.3–27.0)	98.1		
Autumn	73	8701	2671	12.1 (7.2–17.9)	98.5		
Winter	10	912	247	11.2 (1.0–28.6)	99.1		
Unspecified	337	71,530	16,430	13.7 (11.2–16.5)	99.1		
Detecting method						0.0 (0.5954)	< 0.0001
Direct compression	280	44,259	11,169	14.2 (11.4–17.3)	98.9		
Artificial digestion	56	6055	921	18.0 (11.2–25.9)	98.0		
Unspecified	166	42,661	10,257	15.0 (11.3–19.1)	99.2		
*Pseudorasbora* spp.	101	38,086	16,690	49.0 (41.9–56.2)	99.3 (99.2–99.3)		
Period of investigating						18.7 (< 0.0001)	< 0.0001
Before 1990	34	19,663	10,808	65.0 (54.2–75.2)	98.9		
1990–1999	19	4621	2270	59.3 (44.4–73.4)	98.6		
2000–2009	26	5630	2198	42.7 (30.4–55.5)	99.0		
After 2010	22	8172	1414	23.9 (13.2–36.5)	99.0		
Season of investigating						1.8 (0.2113)	< 0.0001
Spring	7	1443	661	68.3 (41.9–89.6)	99.7		
Summer	12	2546	1044	43.3 (23.9–63.8)	99.0		
Autumn	15	4434	2183	46.5 (28.9–64.7)	99.0		
Winter	2	322	244	89.2 (44.8–100.0)	98.8		
Unspecified	65	29,341	12,558	47.1 (38.4–56.0)	99.3		
Detecting method						0.9 (0.2383)	< 0.0001
Direct compression	59	21,396	8550	44.1 (35.0–53.4)	99.4		
Artificial digestion	7	543	302	62.5 (35.1–86.3)	95.4		
Unspecified	35	16,147	7838	54.8 (42.7–66.5)	98.8		

*CI* confidence interval, *QM* the regression model heterogeneity, *QE* the residual error heterogeneity

The taxonomic class of the second intermediate host explained the highest level of heterogeneity (*R*^2^ = 35.5, QM = 544.23, *P* < 0.0001; see Table 2). *C.* *sinensis* infections have been reported in several classes of freshwater fishes, including Cyprinidae, Cobitidae, Cichlidae, Eleotridae, and others. Among the Cyprinidae family, *Pseudorasbora* spp. (Cyprinidae: Gobioninae) is the most important second intermediate host of *C.* *sinensis*, with an estimated pooled prevalence of 48.5% (95% *CI*: 44.2–52.7%). Notably, infections in some economic fish species are also common. The pooled prevalence in *Ctenopharyngodon* (the most common fish used to make sashimi in south China) was estimated to be 15.2% (95% *CI*: 10.9–20.1%), while the estimated pooled prevalence in *Cyprinus* spp. (usually used to make raw fish products in northeast China) was 6.1% (95% *CI*: 2.9–10.2%). Besides fishes, infections in shellfish, including shrimp and crab, were also reported, with a pooled prevalence of 0.7% (95% *CI*: 0.0–3.3%).

When stratified by time period of investigation, pooled infection rates decreased significantly after 2010, from 18.2% (95% *CI*: 14.9–21.6%) before 1990 to 10.2% (95% *CI*: 7.8–12.8%) after 2010 (*P* value was 0.0002 in subgroup analysis; see Table 2). When analyzed according to infection level in humans, the pooled prevalence in all second intermediate hosts decreased significantly over time, from 20.3% (15.6–25.3%) before 1990 to 8.8% (5.6–12.6%) after 2010 in low epidemic areas (*R*^2^ = 3.5, *P* = 0.0002). However, such decline was not observed in high epidemic areas, with pooled prevalence of 15.3% (11.2–19.9%) before 1990 to 11.9% (8.6–15.6%) after 2010 (*R*^2^ = 0.0, *P* = 0.6575; see Table 2, Additional file 13: Table S5, Fig. 2).

Moreover, the season could also explain the heterogeneity significantly (*R*^2^ = 2.3, *P* < 0.0001; see Table 2). The infection rate was highest in spring (29.2%, 95% *CI*: 22.4–36.6%). However, it needs to be cautious in explaining the seasonal difference since the overwhelming majority of the studies did not record the season of investigation. Infection rates were similar in subgroups of diverse detecting methods (*R*^2^ = 0.0, *P* = 0.6348).

When analyzed separately, the pooled infection rates of *Pseudorasbora* spp. decreased from 58.2% (95% *CI*: 48.9–67.3%) before 1990 to 32.7% (95% *CI*: 22.3–44.1%) after 2010 (*P* = 0.0057). Similarly, the pooled prevalence in *Pseudorasbora* spp. decreased significantly over time in low epidemic areas, from 65.0% (54.2–75.2%) before 1990 to 23.9% (13.2–36.5%; *R*^2^ = 18.7, *P* < 0.0001) after 2010, while such decline was not observed in high epidemic areas, from 44.8% (29.7–60.3%) to 52.4% (32.9–71.5%; *R*^2^ = 0.0, *P* = 0.8666. See Table 2, Additional file 13: Table S5, Fig. 2).

### *C. sinensis* infection in animal reservoirs

A total of 114 articles containing 239 data points and 60,817 samples of animal reservoirs were included in the meta-analysis. *C.* *sinensis* infections have been reported in cat, dog, fox, yellow weasel, hog badger, rat, pig, cattle, duck, chicken, and crocodile (see Additional file 12: Table S4). The pooled prevalence was estimated to be 14.3% (95% *CI*: 11.4–17.6%), and *I*^*2*^ value was 98.3% (*P* < 0.0001, see Table 3; the forest plot is shown in Additional file 3: Fig. S3). Categories of reservoir animals explained the highest level of heterogeneity (*R*^2^ = 34.9, QM = 129.81, *P* < 0.0001; see Table 3, Additional file 13: Table S5).

**Table 3:** Estimates of pooled prevalence and subgroup analysis of *Clonorchis* *sinensis* in animal reservoirs

	All areas						
	No. of data points	Sample size	No. of positive	Pooled prevalence, % (95% *CI*)	*I*^*2*^, % (95% *CI*)	*R*^*2*^, % (QM *P* value)	QE *P* value
*All* *animal* *reservoirs*	239	60,817	4868	14.3 (11.4–17.6)	98.3 (98.2–98.4)		
Category						34.9 (< 0.0001)	< 0.0001
Cat	52	4911	2040	41.4 (34.0–48.9)	96.6		
Dog	80	17,013	1978	19.5 (14.9–24.6)	98.0		
Pig	61	18,540	530	4.6 (1.9–8.1)	95.0		
Fox	1	240	51	21.3 (0.0–71.1)	0.0		
Yellow weasel	1	28	2	7.1 (0.0–56.2)	0.0		
Hog badger	1	28	2	7.1 (0.0–56.2)	0.0		
Rat	12	3315	139	3.6 (0.0–11.8)	96.4		
Cattle	11	1954	52	0.8 (0.0–7.1)	50.9		
Sheep	1	42	0	0.0 (0.0–32.7)	0.0		
Rabbit	2	13,232	15	0.0 (0.0–16.3)	0.0		
Duck	9	782	49	1.3 (0.0–9.3)	95.7		
Chicken	5	339	1	0.1 (0.0–9.6)	0.0		
Goose	2	65	0	0.0 (0.0–19.7)	0.0		
Crocodile	1	328	9	2.7 (0.0–40.4)	NE		
Period of investigating						1.3 (0.1169)	< 0.0001
Before 1990	83	25,352	1733	16.5 (11.4–22.4)	98.4		
1990–1999	57	14,590	1066	16.2 (10.1–23.3)	98.6		
2000–2009	60	12,888	1415	15.2 (9.5–22.0)	98.4		
After 2010	39	7987	654	7.1 (2.5–13.6)	96.4		
Detecting method						2.1 (0.1004)	< 0.0001
Necropsy examination	85	33,637	2558	20.8 (15.3–27.0)	99.0		
Stool examination: Kato-Katz	19	1939	132	12.9 (4.2–24.9)	96.0		
Stool examination: direct smear	13	4103	243	12.6 (3.0–27.1)	98.6		
Stool examination: sedimentation	26	2458	238	9.7 (3.2–18.8)	94.7		
Stool examination: floating	5	1644	56	3.6 (0.0–21.2)	28.5		
Stool examination	58	11,471	751	10.0 (5.2–15.9)	96.9		
Unspecified	33	5565	890	14.8 (7.5–23.8)	97.9		
*Dogs*	80	17,013	1978	19.6 (14.6–25.0)	98.0 (97.8–98.2)		
Period of investigating						27.7 (< 0.0001)	< 0.0001
Before 1990	27	2978	921	32.6 (24.0–41.9)	92.3		
1990–1999	11	619	245	32.6 (19.0–47.7)	96.8		
2000–2009	23	9462	641	15.4 (8.5–23.6)	98.4		
After 2010	19	3954	171	5.0 (1.0–11.3)	90.3		
Detecting method						23.6 (0.0001)	< 0.0001
Necropsy examination	24	2574	842	36.5 (26.7–46.9)	93.6		
Stool examination: Kato-Katz	8	710	23	5.5 (0.0–17.2)	81.6		
Stool examination: direct smear	5	3485	45	7.4 (0.0–23.4)	95.9		
Stool examination: sedimentation	9	1098	103	13.2 (3.8–26.8)	92.4		
Stool examination: floating	1	118	5	4.2 (0.0–42.3)	28.5		
Stool examination	25	7580	541	13.1 (6.9–20.8)	97.7		
Unspecified	8	1448	419	33.0 (17.3–50.9)	94.4		
*Cats*	52	4911	2040	41.4 (33.3–49.6)	96.6 (96.1–97.1)		
Period of investigating						5.5 (0.1331)	< 0.0001
Before 1990	12	794	379	50.1 (33.5–66.8)	92.0		
1990–1999	13	1095	545	44.0 (28.5–60.0)	95.5		
2000–2009	18	1574	750	44.0 (30.7–57.8)	96.3		
After 2010	9	1448	366	22.6 (8.8–40.3)	97.7		
Detecting method						17.2 (0.0138)	< 0.0001
Necropsy examination	19	2165	1216	57.4 (44.8–69.4)	94.5		
Stool examination: Kato-Katz	3	529	44	13.6 (0.1–40.7)	92.9		
Stool examination: direct smear	5	454	198	35.0 (14.3–59.0)	97.0		
Stool examination: sedimentation	5	254	67	34.4 (13.6–58.9)	94.9		
Stool examination	13	599	158	28.0 (15.3–42.8)	92.7		
Unspecified	7	910	357	46.7 (27.2–66.7)	93.2		

	Areas with population infection rate ≥ 1.0%						
	No. of data points	Sample size	No. of positive	Pooled prevalence, % (95% *CI*)	*I*^*2*^, % (95% *CI*)	*R*^*2*^, % (QM *P* value)	QE *P* value
*All* *animal* *reservoirs*	86	8533	1885	16.5 (11.0–22.8)	98.2 (98.0;98.4)		
Category						68.7 (< 0.0001)	< 0.0001
Cat	23	2356	1321	52.8 (43.9–61.7)	94.0		
Dog	26	1915	513	27.4 (20.2–35.3)	93.9		
Pig	19	1326	23	0.5 (0.0–3.6)	72.7		
Fox							
Yellow weasel							
Hog badger							
Rat	6	2136	28	0.4 (0.0–6.0)	94.2		
Cattle	5	212	0	0.0 (0.0–6.2)	0.0		
Sheep							
Rabbit							
Duck	5	512	0	0.0 (0.0–5.1)	0.0		
Chicken	1	41	0	0.0 (0.0–22.4)	NE		
Goose	1	35	0	0.0 (0.0–23.2)	NE		
Crocodile							
Period of investigating						0.0 (0.4679)	< 0.0001
Before 1990	22	1142	179	11.7 (3.2–23.9)	93.3		
1990–1999	25	3715	550	12.8 (4.4–24.2)	98.9		
2000–2009	31	2820	846	22.4 (12.3–34.4)	97.9		
After 2010	8	856	310	20.8 (4.0–45.1)	97.0		
Detecting method						5.9 (0.0736)	< 0.0001
Necropsy examination	51	4798	1490	18.9 (11.6–27.4)	97.9		
Stool examination: Kato-Katz	1	35	3	8.6 (0.0–73.3)	NE		
Stool examination: direct smear	10	753	218	16.9 (3.5–36.7)	97.9		
Stool examination: sedimentation	5	245	55	17.9 (0.8–47.4)	95.4		
Stool examination: floating							
Stool examination	17	2645	76	5.7 (0.1–16.7)	95.1		
Unspecified	2	57	43	75.8 (26.1–100.0)	76.0		
*Dogs*	26	1915	513	27.5 (18.7–37.2)	93.9 (92.1–95.3)		
Period of investigating						16.1 (0.0854)	< 0.0001
Before 1990	8	447	102	22.6 (10.0–38.3)	69.0		
1990–1999	4	222	144	55.8 (31.5–78.7)	96.3		
2000–2009	11	1148	249	24.7 (13.2–38.3)	91.6		
After 2010	3	98	18	17.3 (1.3–43.4)	88.5		
Detecting method						19.1 (0.0670)	< 0.0001
Necropsy examination	15	1274	379	30.4 (19.6–42.3)	92.7		
Stool examination: Kato-Katz	1	35	3	8.6 (0.0–50.7)	NE		
Stool examination: direct smear	3	220	27	14.5 (0.9–38.3)	96.6		
Stool examination: sedimentation	2	127	38	34.5 (8.1–67.3)	93.5		
Stool examination: floating							
Stool examination	4	229	40	17.0 (3.1–38.0)	75.7		
Unspecified	1	30	26	86.7 (41.3–100.0)	NE		
*Cats*	23	2356	1321	52.6 (41.3–63.9)	94.0 (92.2–95.4)		
Period of investigating						0.0 (0.9964)	< 0.0001
Before 1990	3	119	61	52.6 (20.2–83.9)	0.0		
1990–1999	6	586	371	50.9 (27.7–73.9)	95.8		
2000–2009	11	1008	597	54.1 (36.4–71.3)	94.4		
After 2010	3	643	292	50.8 (19.3–81.8)	89.6		
Detecting method						0.0 (0.9010)	< 0.0001
Necropsy examination	14	1849	1060	56.6 (41.0–71.5)	94.9		
Stool examination: Kato-Katz							
Stool examination: direct smear	4	369	191	43.6 (17.2–72.0)	95.2		
Stool examination: sedimentation	1	33	17	51.5 (3.5–97.7)	NE		
Stool examination	3	78	36	42.4 (12.0–76.3)	94.8		
Unspecified	1	27	17	63.0 (8.9–100.0)	NE		

	Areas with population infection rate < 1.0%						
	No. of data points	Sample size	No. of positive	Pooled prevalence, % (95% *CI*)	*I*^*2*^, % (95% *CI*)	*R*^*2*^, % (QM *P* value)	QE *P* value
*All* *animal* *reservoirs*	153	52,284	2983	13.2 (9.9–16.9)	98.1 (98.0–98.2)		
Category						15.4 (0.0003)	< 0.0001
Cat	29	2555	719	32.8 (23.1–43.3)	95.8		
Dog	54	15,098	1465	16.2 (10.7–22.4)	98.3		
Pig	42	17,214	507	7.3 (3.2–12.7)	96.4		
Fox	1	240	51	21.3 (0.0–74.2)	NE		
Yellow weasel	1	28	2	7.1 (0.0–59.6)	NE		
Hog badger	1	28	2	7.1 (0.0–59.6)	NE		
Rat	6	1179	111	9.5 (0.3–27.5)	90.0		
Cattle	6	1742	52	2.1 (0.0–13.7)	59.3		
Sheep	1	42	0	0.0 (0.0–36.2)	NE		
Rabbit	2	13,232	15	0.0 (0.0–18.4)	0.0		
Duck	4	270	49	5.4 (0.0–25.7)	97.7		
Chicken	4	298	1	0.1 (0.0–12.9)	0.0		
Goose	1	30	0	0.0 (0.0–38.3)	NE		
Crocodile	1	328	9	2.7 (0.0–44.1)	NE		
Period of investigating						7.4 (0.0024)	< 0.0001
Before 1990	61	24,210	1554	18.3 (12.7–24.7)	98.8		
1990–1999	32	10,875	516	19.0 (11.2–28.2)	98.1		
2000–2009	29	10,068	569	8.9 (3.4–16.4)	97.8		
After 2010	31	7131	344	4.7 (1.0–10.4)	90.5		
Detecting method						3.3 (0.0864)	< 0.0001
Necropsy examination	34	28,839	1068	23.7 (15.3–33.2)	99.0		
Stool examination: Kato-Katz	18	1904	129	13.1 (4.6–24.6)	96.2		
Stool examination: direct smear	3	3350	25	2.9 (0.0–25.0)	90.9		
Stool examination: sedimentation	21	2213	183	8.0 (2.0–17.0)	94.4		
Stool examination: floating	5	1644	56	3.6 (0.0–19.7)	28.5		
Stool examination	41	8826	675	12.0 (6.3–19.1)	97.0		
Unspecified	31	5508	847	12.0 (5.7–20.1)	97.5		
*Dogs*	54	15,098	1465	16.2 (10.6–22.5)	98.3 (98.1–98.5)		
Period of investigating						43.1 (< 0.0001)	< 0.0001
Before 1990	19	2531	819	37.0 (27.3–47.2)	93.9		
1990–1999	7	397	101	20.5 (7.9–36.6)	95.0		
2000–2009	12	8314	392	8.5 (2.5–17.3)	98.7		
After 2010	16	3856	153	3.5 (0.4–9.1)	89.7		
Detecting method						30.5 (0.0001)	< 0.0001
Necropsy examination	9	1300	463	46.8 (30.8–63.1)	94.8		
Stool examination: Kato-Katz	7	675	20	5.1 (0.0–17.1)	82.5		
Stool examination: direct smear	2	3265	18	1.3 (0.0–19.4)	79.5		
Stool examination: sedimentation	7	971	65	8.6 (0.9–22.0)	85.8		
Stool examination: floating	1	118	5	4.2 (0.0–40.9)	NE		
Stool examination	21	7351	501	12.4 (6.1–20.5)	98.0		
Unspecified	7	1418	393	26.0 (11.3–43.8)	92.1		
*Cats*	29	2555	719	32.8 (23.1–43.3)	95.8 (94.8–96.6)		
Period of investigating						23.0 (0.0163)	< 0.0001
Before 1990	9	675	318	49.3 (32.4–66.4)	94.0		
1990–1999	7	509	174	38.0 (20.3–57.5)	87.5		
2000–2009	7	566	153	29.1 (13.3–47.8)	94.2		
After 2010	6	805	74	11.5 (1.6–27.8)	88.9		
Detecting method						18.9 (0.0507)	< 0.0001
Necropsy examination	5	316	156	59.5 (35.7–81.2)	94.1		
Stool examination: Kato-Katz	3	529	44	13.6 (0.2–39.6)	92.9		
Stool examination: direct smear	1	85	7	8.2 (0.0–52.4)	NE		
Stool examination: sedimentation	4	221	50	30.4 (9.5–56.5)	95.3		
Stool examination	10	521	122	24.2 (11.3–39.9)	91.9		
Unspecified	6	883	340	44.2 (24.4–64.9)	93.8		

*CI* confidence interval, *QM* the regression model heterogeneity, *QE* the residual error heterogeneity

The pooled prevalence of *C.* *sinensis* was highest in cat, with a prevalence of 41.4% (95% *CI*: 34.0–48.9%). Infections in other animals were also common, including dog (19.5%, 95% *CI*: 14.9–24.6%), pig (4.6%, 95% *CI*: 1.9–8.1%), and rat (3.6%, 95% *CI*: 0.0–11.8%). These findings suggest that *C.* *sinensis* infections are not limited to a specific animal species and may be present in a variety of animals.

The overall pooled prevalence in animal reservoirs decreased over time, from 16.5% (11.4–22.4%) before 1990 to 7.1% (2.5–13.6%) after 2010 (*P* value was 0.0008 in multivariable meta-regression model; see Table 3, Additional file 13: Table S5). When analyzed according to infection level in humans, the pooled prevalence in animal reservoirs decreased significantly, from 18.3% (12.7–24.7%) before 1990 to 4.7% (1.0–10.4%) after 2010 in low epidemic areas (*R*^2^ = 7.4, *P* = 0.0024); however, such decline was not observed in high epidemic areas, with pooled prevalence (95% *CI*) of 11.7% (3.2–23.9%) to 20.8% (4.0–45.1%; *R*^2^ = 0.0, *P* = 0.4679; see Table 3, Additional file 13: Table S5, Fig. 2).

In addition, the detecting method can also partially explain the heterogeneity when analyzed separately for cats (*R*^2^ = 17.2, *P* = 0.0138) and dogs (*R*^2^ = 23.6, *P* = 0.0001). Compared with various stool examinations, necropsy examination reported a higher pooled prevalence in both cats (57.4%, 95% *CI*: 44.8–69.4%) and dogs (36.5%, 95% *CI*: 26.7–46.9%).

When analyzed separately, the pooled prevalence (95% *CI*) of *C.* *sinensis* infection in high epidemic areas showed a significant decrease in cats, from 49.3% (32.4–66.4%) before 1990 to 11.5% (1.6–27.8%) after 2010 (*R*^2^ = 23.0, *P* = 0.0163), and in dogs, from 36.9% (27.3–47.2%) to 3.5% (0.4–9.1%) (*R*^2^ = 43.1, *P* < 0.0001). However, such decline was not observed in low epidemic areas, with the pooled prevalence remaining relatively stable in cats, from 52.6% (20.2–83.9%) before 1990 to 50.8% (19.3–81.8%) after 2010 (*R*^2^ = 0.0, *P* = 0.9964), and in dogs, from 22.6% (10.0–38.3%) to 17.3% (1.3–43.4%) (*R*^2^ = 16.1, *P* = 0.0854; see Table 3, Additional file 13: Table S5, Fig. 2). Moreover, the results of multivariable meta-regression model further verified the spatial–temporal disparities both in cats and in dogs (Additional file 13: Table S5).

### Publication bias and sensitivity analysis

The presence of publication bias was detected through funnel plots, and the result of Egger's test revealed the potential existence of publication bias (see Additional file 4: Fig. S4a, Additional file 5: Fig. S4b, Additional file 6: Fig. S4c). However, sensitivity analysis demonstrated that the pooled prevalence did not change significantly when excluding outliers, removing data with small sample sizes, or excluding studies without reporting the detecting method to measure *C.* *sinensis* infection (i.e., studies with moderate or high risk of bias). The 95% *CI* remained overlapping, indicating the robustness of the main results (see Additional file 14: Table S6). Additionally, similar temporal disparities were also observed in both low and high epidemic areas. This suggests that the temporal trends in *C.* *sinensis* infection rates remained consistent even after accounting for the quality of publications (see Additional file 15: Table S7).

### Spatio-temporal distribution and biogeographical characteristics of *C. sinensis* infection in animal hosts

**Fig. 3:**
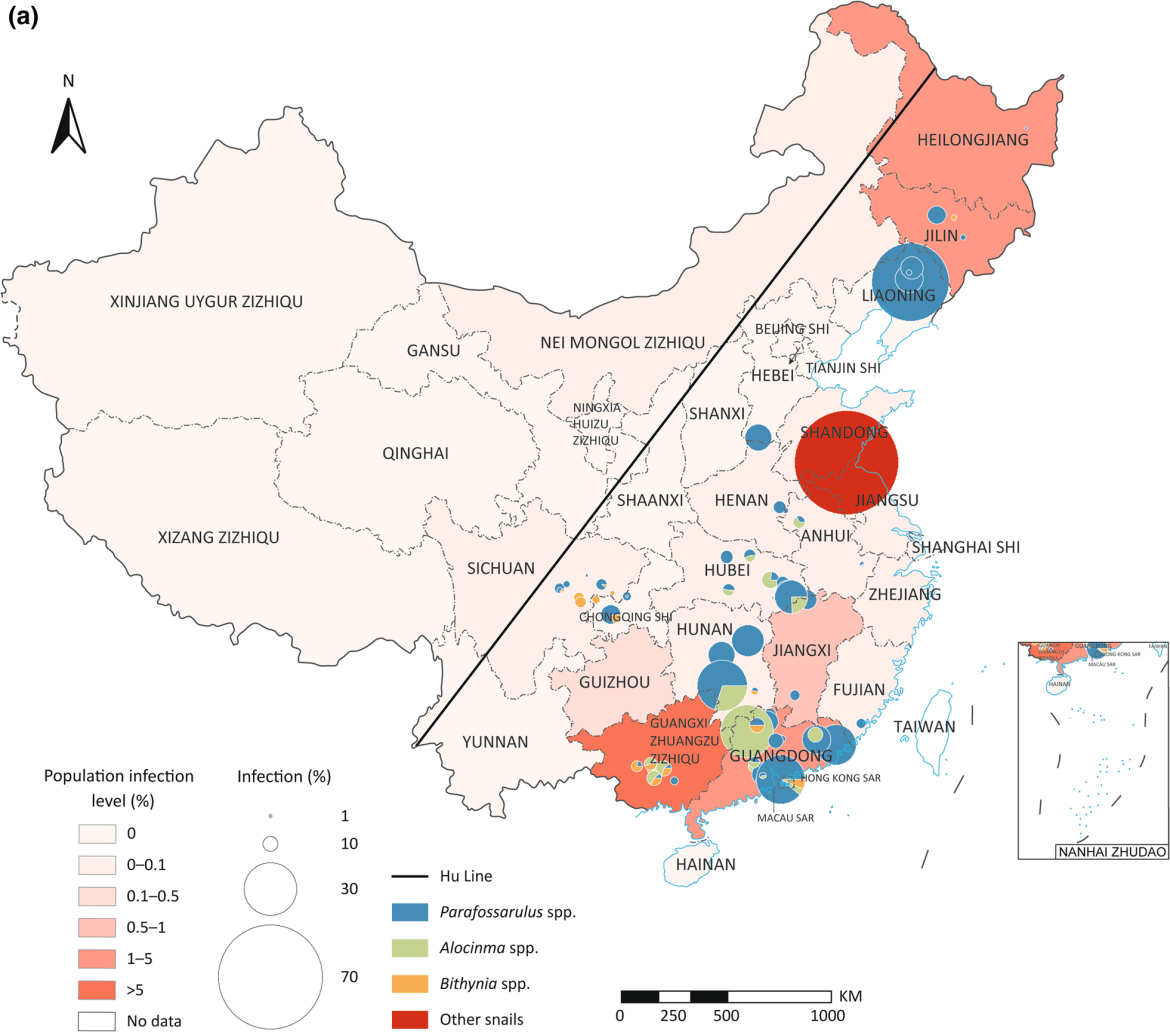

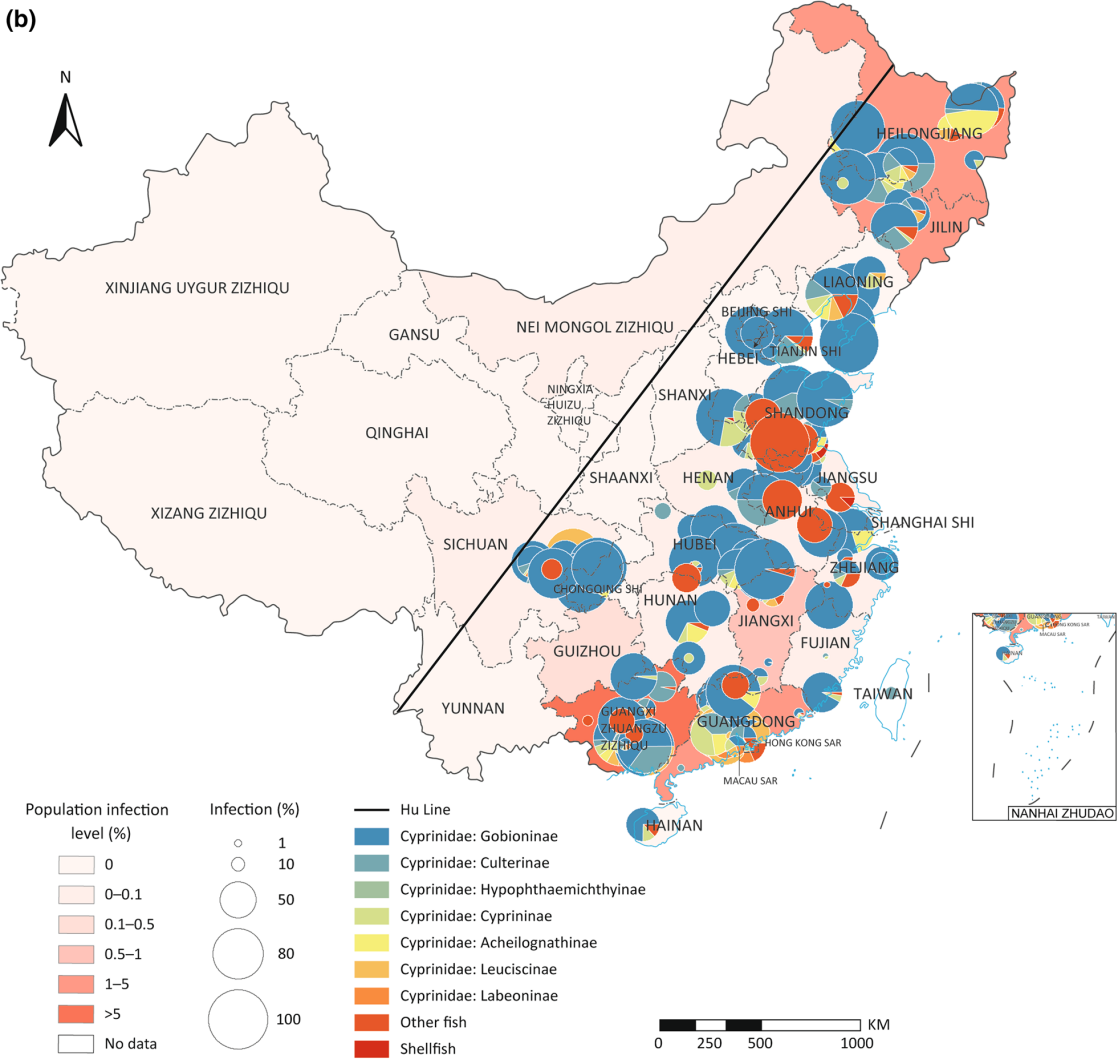

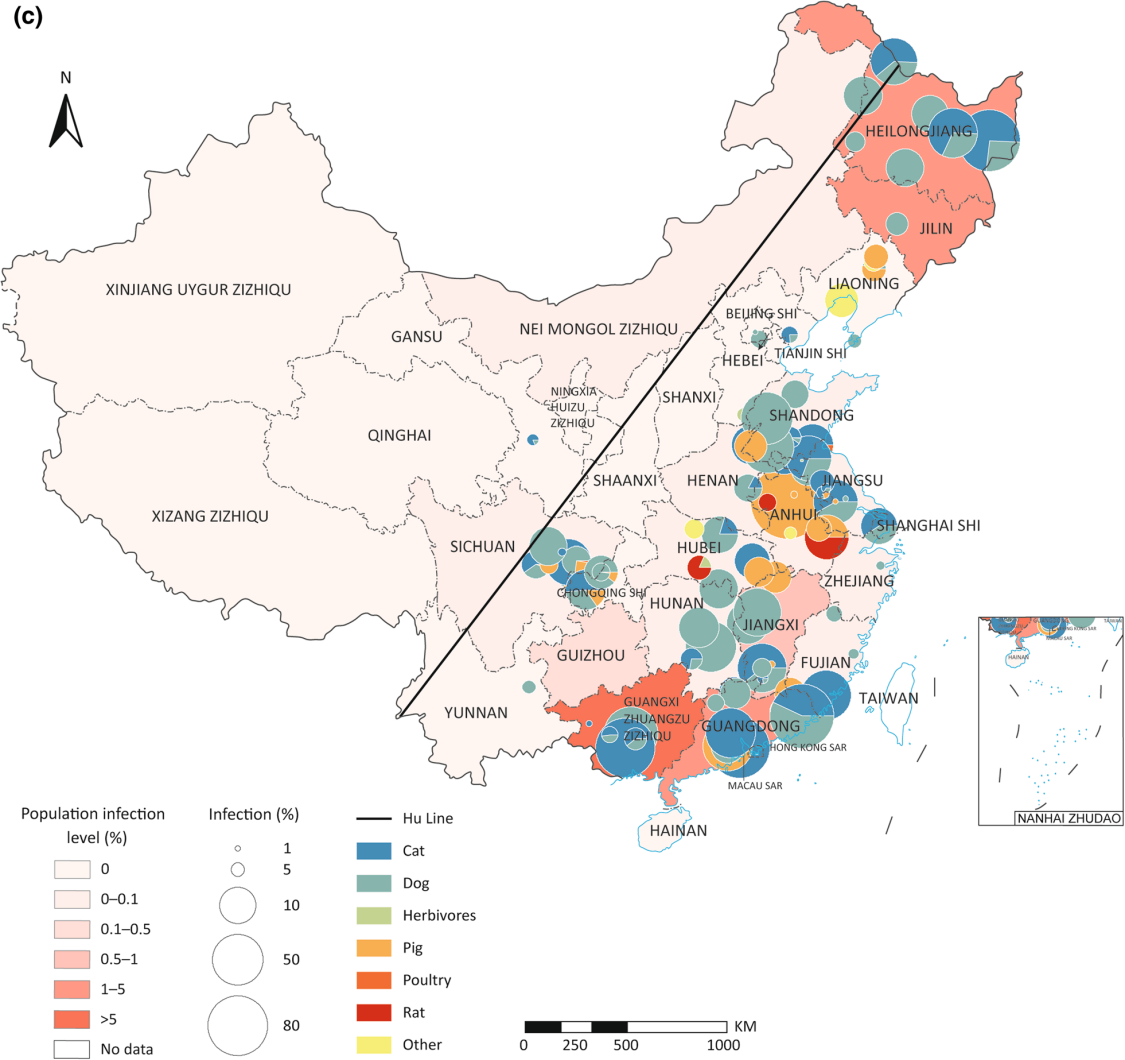
Distribution and prevalence of *Clonorchis* *sinensis* infection in diverse animal hosts in China. **a** *C.* *sinensis* in snails; **b** *C.* *sinensis* in second intermediate hosts; **c** *C.* *sinensis* in animal reservoirs. The diagonal lines in all maps are the Heihe-Tengchong Line (Hu Line)

A total of 114 survey sites of first intermediate hosts, 223 s intermediate hosts, and 123 animal reservoirs were geographically referenced and plotted on the epidemic map of China. Infections of *C.* *sinensis* in animal hosts in China were predominantly reported in areas east of the Heihe-Tengchong Line (Hu Line) [36], which roughly corresponds to the 400 mm precipitation line of China (see Fig. 3). For the first intermediate hosts, *Parafossarulus* spp. infections were reported widely, while *Alocinma* spp. and *Bithynia* spp. infections were mainly reported in the south areas (Fig. 3a); for the second intermediate hosts, infections in fishes of Cyprinidae, especially Gobioninae, were reported most widely (Fig. 3b); while for animal reservoirs, infections in cats and dogs were widely distributed (Fig. 3c). Interestingly, the infection rates in animal hosts were not consistent with the epidemic levels of human *C.* *sinensis* infection. For instance, high infection rates in snails, second intermediate hosts, or animal reservoirs were frequently reported in low-endemic PLADs of China (see Fig. 3).

**Fig. 4:**
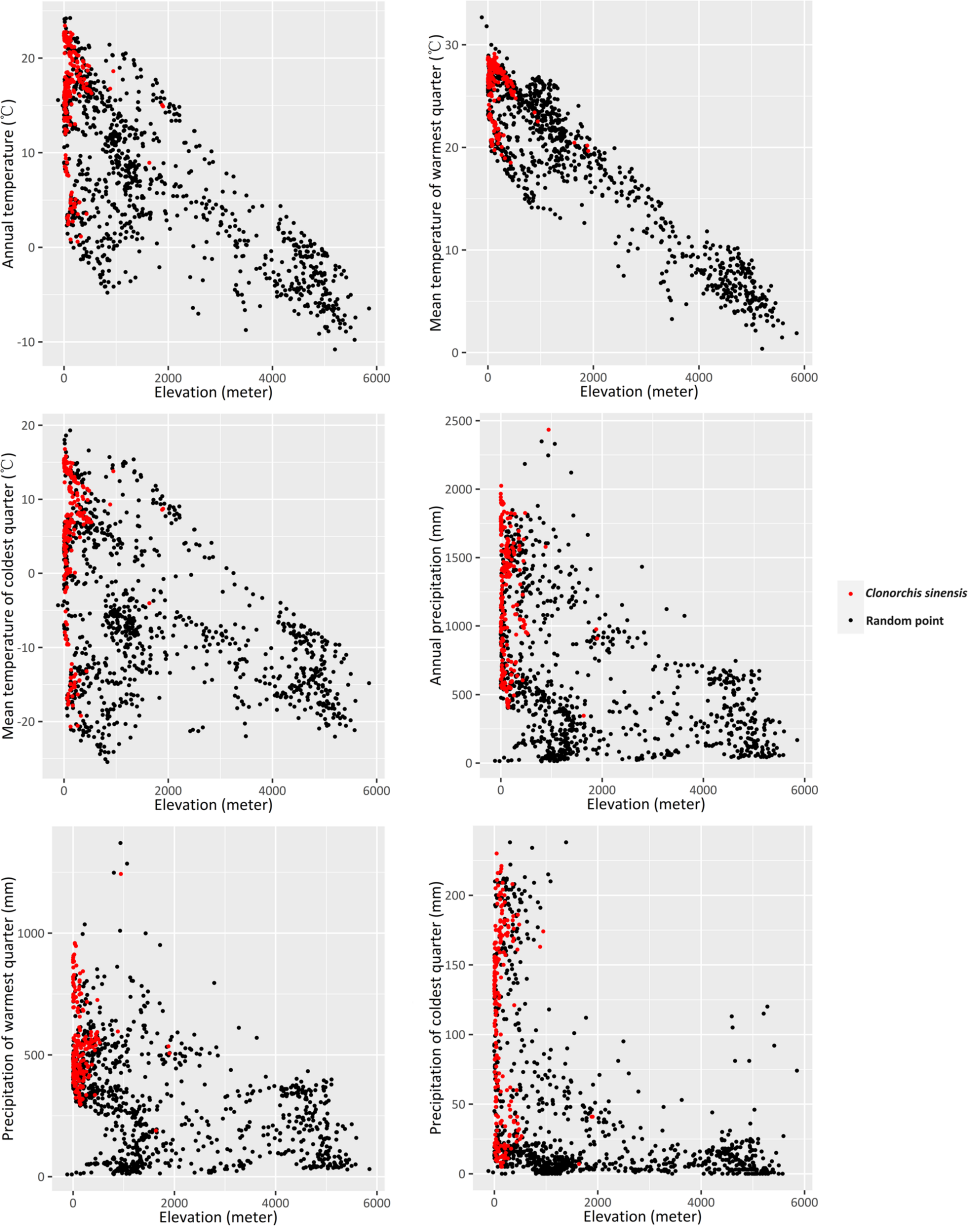
Environmental characteristics of regions reporting animal *Clonorchis* *sinensis* infections in China

To explore the biogeographical characteristics of *C.* *sinensis* infection in animals, we used scatter plots to display the environmental dimensions. The results, depicted in Fig. 4 and summarized in Table 4, indicate that animals with *C.* *sinensis* infection are predominantly reported in areas with specific environmental conditions. These conditions include an annual mean temperature above − 0.24 °C, a mean temperature of the warmest quarter above 16.21 °C, an annual precipitation above 345 mm, a precipitation of the warmest quarter above 189 mm, and an altitude below 2346 m.

**Table 4:** Environmental characteristics of regions reporting *Clonorchis* *sinensis* infection in animals

Variable	Min–Max	Percentile (0.05–0.95)	Mean ± *SD*
*The* *first* *intermediate* *host*			
Elevation (meter)	− 1.00–527.00	3.00–479.00	142.06 ± 142.23
Annual mean temperature (°C)	3.11–22.73	4.26–22.73	17.20 ± 4.94
Mean temperature of warmest quarter (°C)	20.38–28.82	21.09–28.58	26.74 ± 2.08
Mean temperature of coldest quarter (°C)	− 16.88–15.83	− 15.33–15.45	6.51 ± 8.26
Annual precipitation (mm)	435.00–2024.00	516.00–1908.00	1289.64 ± 421.17
Precipitation of warmest quarter (mm)	310.00–959.00	325.20–947.00	570.39 ± 161.73
Precipitation of coldest quarter (mm)	5.00–217.00	10.00–192.00	111.87 ± 62.89
*The* *second* *intermediate* *host*			
Elevation (meter)	− 2.00–2346.00	3.00–479.00	109.87 ± 144.94
Annual mean temperature (°C)	− 0.24–24.53	4.26–22.73	16.05 ± 5.99
Mean temperature of warmest quarter (°C)	16.21–29.08	21.09–28.58	26.26 ± 2.50
Mean temperature of coldest quarter (°C)	− 20.74–19.40	− 15.33–15.45	4.64 ± 9.96
Annual precipitation (mm)	364.00–2434.00	516.00–1908.00	1197.82 ± 476.68
Precipitation of warmest quarter (mm)	226.00–1243.00	325.20–947.00	547.18 ± 179.82
Precipitation of coldest quarter (mm)	3.00–219.00	10.00–192.00	101.45 ± 65.29
*Reservoir* *hosts*			
Elevation (meter)	− 1.00–2045.00	5.00–457.00	153.15 ± 211.3
Annual mean temperature (°C)	0.59–22.69	4.54–22.48	16.77 ± 4.92
Mean temperature of warmest quarter (°C)	18.51–28.78	20.45–28.74	26.54 ± 2.19
Mean temperature of coldest quarter (°C)	− 20.69–15.45	− 14.11–14.82	5.89 ± 8.08
Annual precipitation (mm)	345.00–2024.00	559.00–1724.00	1212.56 ± 426.26
Precipitation of warmest quarter (mm)	189.00–907.00	346.00–827.00	538.48 ± 142.42
Precipitation of coldest quarter (mm)	5.00–221.00	16.00–203.00	106.43 ± 66.57

*SD* standard deviation, *Min* minimum, *Max* maximum

## Discussion

*C.* *sinensis* infection represents a substantial global health threat, with over 200 million people estimated to be at risk of infection worldwide. Among this vulnerable population, more than 35 million individuals are currently affected, with approximately 1.5–2 million experiencing symptoms or complications [7, 37, 38]. Recognizing the significance of zoonotic diseases, the concept of One Health has gained increasing recognition as a critical approach to disease control. This integrated approach underscores the interconnectedness between humans, animals, and the environment, playing a pivotal role in safeguarding the health of humans, animals, and the ecosystems within the animal food-supply chain [39, 40]. Understanding the prevalence of infection in animal hosts is crucial in our efforts to effectively control human clonorchiasis in this complex scenario.

In China, the findings of three national parasite surveys have revealed regional disparities in the temporal trends of *C.* *sinensis* infection in human populations [41]. While infection rates have significantly declined in most regions of China, they have remained persistently high in four PLADs: Guangxi, Guangdong, Heilongjiang, and Jilin, where the infection rate exceeds 1.0% [42]. However, our understanding of the spatio-temporal trends of *C.* *sinensis* infection in different animal hosts remains limited. To address this gap in knowledge, we undertook a comprehensive systematic review and meta-analysis, synthesizing data from various studies on *C.* *sinensis* infections in diverse animal hosts. Our study aimed to provide valuable insights into the spatio-temporal distribution and biogeographical patterns of *C.* *sinensis* infections in animal hosts across China.

Our study revealed that at least eight species of freshwater snails, namely *P.* *striatulus*, *P.* *sinensis*, *P.* *anomalospiralis*, *A.* *longicornis*, *B.* *fuchslana*, *B.* *robust*, *B.* *misella*, and *S.* *cancellata*, can serve as first intermediate hosts of *C.* *sinensis* in China. These snails are commonly found in environments with a suitable climate, characterized by cool and slow-moving water bodies such as streams, lakes, ponds, marshes, paddy fields, and small ditches [10]. *P.* *striatulus*, *A.* *longicornis*, and *B.* *fuchsianus* were found to have wide distributions in the eastern regions of China (Additional file 7: Fig. S5), and these species were identified as major transmission vectors of *C.* *sinensis*. This highlights the importance of monitoring snail populations in regions with endemic clonorchiasis to gain better insights into the transmission dynamics of the parasite.

The second intermediate hosts of *C.* *sinensis* include freshwater fish and shellfish, with freshwater fish being particularly relevant to human infection [43]. The variation in prevalence in these hosts was significantly influenced by the taxonomic class of the second intermediate host. Fishes in the Cypriniformes order are considered to be the most common second intermediate host of *C.* *sinensis* [44]. Previous laboratory transmission experiments indicated that *P.* *parva* was more susceptible to *C.* *sinensis* compared to other fish species [45]. Similarly, our study identified *Pseudorasbora* spp. as the most commonly reported fish species infected with *C.* *sinensis*, showing a high overall pooled infection rate of 48.5% (95% *CI*: 44.2–52.7%) compared to other fish species. *Pseudorasbora* spp. is an invasive freshwater fish species known for its ubiquity, sedentary nature, hardiness, and omnivorous diet. It can be found in almost all natural and man-made water bodies throughout China [46, 47]. Additionally, *Pseudorasbora* spp. is often used as feed for animals after being caught [48], suggesting its potential role in maintaining the lifecycle of *C.* *sinensis* in China.

Infections in some major aquaculture fish species in China were also prevalent [49]. For instance, the overall pooled infection rate was 15.2% (95% *CI*: 10.9–20.1%) in grass carp (*Ctenopharyngodon* spp.), 6.1% (2.9–10.2%) in *Cyprinus* spp., and 10.9% (95% *CI*: 6.9–15.7%) in *Hemiculter* spp. Both *Ctenopharyngodon* spp. and *Cyprinus* spp. are important aquaculture species not only in China but also in other Southeast Asian countries [50, 51]. Notably, *Ctenopharyngodon* spp. is widely used to prepare sashimi and other raw fish products in southern China, including Guangxi and Guangdong, while *Cyprinus* spp. is commonly used for raw fish products in northeastern China, including Heilongjiang and Jilin. Therefore, it remains crucial to protect water bodies from fecal pollution in order to effectively control the transmission of *C.* *sinensis* in China.

Both the direct compression method and the artificial digestion method are commonly used for examining *C.* *sinensis* metacercariae in freshwater fishes. A study conducted by Li and colleagues compared the detection rates of the two methods using the same fish samples and found no significant difference in detection rate between them [52]. Consistently, our study also found that the pooled infection rates of fish were similar among different detecting method groups in both high and low epidemic areas (Tables 1, 2, 3). Given that the direct compression method is less complicated and less time-consuming compared to the artificial digestion method, we recommend prioritizing its use in examining *C.* *sinensis* metacercariae in fishes during epidemiological surveys. This approach can facilitate efficient data collection and enhance our understanding of the parasite's prevalence in aquatic environments.

Dogs and cats are recognized as the most significant animal reservoirs of *C.* *sinensis* [18]. Their infections are likely attributed to their feeding habits, such as consuming raw fish or the raw entrails of fish [53]. It is worth noting that infections in animals other than carnivores have also been reported, including rats, pigs, ducks, chickens, cattle, rabbits, and crocodiles. However, further research is needed to determine whether these animals act as accidental hosts or play active roles in the transmission of this parasite.

When infections in dogs or cats were analyzed separately, the detection rate of stool examination was found to be lower than that of necropsy examination. This discrepancy can be attributed to the intermittent shedding of parasite eggs, where a single fecal specimen might not capture the presence of eggs, leading to potential false-negative results [54, 55]. As a result, stool examination might underestimate the true infection rate in animal reservoirs. To ensure more accurate and reliable assessments, multiple fecal specimens or alternative diagnostic methods, such as necropsy examination, should be considered in future studies on the prevalence of *C.* *sinensis* in animal reservoirs. Improved diagnostic approaches will enhance our understanding of the role of these animal reservoirs in the transmission of the parasite and aid in the development of effective control measures.

The analyses of biogeographical characteristics of animal infections revealed that temperature significantly influences the distribution of *C.* *sinensis*. Specifically, the temperature during the warmest quarter of the year has a greater impact on the distribution of the parasite than the temperature during the coldest quarter (Fig. 4). The observed association between temperature and the distribution of *C.* *sinensis* in animal hosts can be attributed to several factors.

Firstly, the population dynamics of snails, which serve as important intermediate hosts for *C.* *sinensis*, are often influenced by environmental temperature [56]. For example, previous studies have demonstrated that the peak population of certain snail species, such as *P.* *manchouricus*, begins to occur in April, peaks in June, and disappears after November in specific regions [57]. Moreover, our findings indicate that temperature during the warmest quarter is a more critical factor influencing the distribution of major snail vectors of *C.* *sinensis* in China than the temperature during the coldest quarter (Additional file 8: Fig. S6).

Secondly, temperature also affects the development of larval *C.* *sinensis* within snails. Studies have shown that *C.* *sinensis* infection in snails is most commonly observed during warmer months, and the release of cercariae from infected snails ceases under lower temperatures. For instance, in the study by Chung and colleagues, *C.* *sinensis* infections in *P.* *manchouricus* snails were only observed from May to August in a river in Korea [57]. Additionally, laboratory experiments by Liang et al. revealed that no cercariae were released from infected snails at temperatures below 20 °C [45].

Furthermore, altitude was found to be related to the distribution of *C.* *sinensis* in our study. Animal infections were predominantly reported in areas with altitudes below 2346 m (Fig. 4). However, it is likely that altitude's influence is mediated by its effect on the temperature during the warmest season. As depicted in Fig. 4, altitude shows a nearly perfect linear correlation with the mean temperature of the warmest quarter. This suggests that higher altitudes may experience cooler temperatures during the warmest season, which could impact the prevalence of *C.* *sinensis* in the animal hosts inhabiting these regions.

Our study revealed the distribution of *C.* *sinensis* was also found to be associated with precipitation. In addition, *C.* *sinensis* infections were predominantly reported in eastern China, as depicted in Fig. 3. This geographical pattern corresponds to the known distribution of its primary snail vectors, namely *P.* *striatulus*, *A.* *longicornis*, and *B.* *fuchslana* (Additional file 7: Fig. S5). The distribution of these intermediate hosts is closely linked to water supply, making precipitation a critical driving factor influencing the distribution of both freshwater snails and fishes, which are essential components of the *C.* *sinensis* life cycle. The regions in eastern China are characterized by higher levels of precipitation, which create more suitable and conducive environments for the survival and proliferation of freshwater snails and fishes, consequently increasing the risk of *C.* *sinensis* infections in these areas.

Over the past few decades, there have been significant changes in behaviors and habits related to food consumption and hygiene practices in China, contributing to a decline in the transmission of parasitic diseases [58, 59]. Health awareness and education campaigns have played a crucial role in promoting safe food practices, such as treating raw and cooked foods separately, adopting sanitary toilet facilities, and ensuring access to safe drinking water. Additionally, emphasis on individual hygiene habits has been widely promoted, encouraging proper handwashing and personal cleanliness. Despite the progress made in controlling parasitic diseases in China, certain regions continue to face challenges due to persistent habits of consuming raw animal foods [60–62]. This behavior contributes to the ongoing prevalence of food-borne parasitic diseases, including *C.* *sinensis* infections. For example, while the infection rate of *C.* *sinensis* in humans has significantly decreased in most areas of the country, it remains stubbornly high in PLADs like Guangxi, Guangdong, Heilongjiang, and Jilin [17]. In regions where the human prevalence of clonorchiasis is less than 1.0%, we observed a significant decline in the infection rate of *C.* *sinensis* in animal hosts after 2010 (see Tables 1, 2, 3, Fig. 2), which can be attributed to successful control measures targeting humans, animal hosts, and the environment. These measures include improved sanitation practices, changes in food preparation habits, and health education programs [59]. However, in areas with higher human prevalence, the infection rate in animal hosts remains consistently high. It suggests that despite efforts to control human clonorchiasis, the transmission of *C.* *sinensis* from animal hosts to humans continues to occur.

To effectively reduce the prevalence of *C.* *sinensis* in both human and animal populations, these high-prevalence regions may require additional interventions and comprehensive measures. Implementing targeted strategies that address the specific transmission dynamics in these areas, as well as promoting the concept of One Health, which recognizes the interconnectedness of human, animal, and environmental health, could prove crucial in breaking the transmission cycle of the parasite. Continued surveillance, health education, and collaboration between public health, veterinary, and environmental authorities are essential for sustained progress in controlling *C.* *sinensis* infections and other food-borne parasitic diseases in China.

The study indeed has several limitations that should be acknowledged. One major limitation is the uneven distribution of studies across different regions in China. This uneven distribution may introduce bias in the pooled estimates of infections, as the data may not be fully representative of the entire country. This limitation should be taken into account when interpreting the findings. Secondly, although we found that environmental factors, such as temperature, had an impact on the existence of *C.* *sinensis*, we did not analyze how such factors influence the prevalence of *C.* *sinensis* in animals. The reason for this lies in the unavailability of survey time for the majority of the included publications, preventing us from obtaining information on environmental factors, such as temperature, at the time each study was conducted.

Furthermore, like many meta-analyses on prevalence, the presence of publication bias is a concern in our study [23, 63, 64]. This bias can distort the estimates of prevalence and may affect the overall conclusions. Although we conducted sensitivity analysis and examined funnel plots, the potential impact of publication bias should be considered when interpreting the results. In addition, the heterogeneity observed in some analyses may also introduce uncertainty into the findings. Heterogeneity can arise from variations in study designs, populations, methodologies, and other factors across the included studies. While we used random-effects models to account for heterogeneity, it may still influence the overall precision and reliability of the pooled estimates.

Despite these limitations, our study provides valuable insights into the spatio-temporal distribution and biogeographical patterns of *C.* *sinensis* infections in animal hosts across China. By acknowledging these limitations, researchers and readers can have a more comprehensive understanding of the study's findings and the potential implications of the results.

## Conclusions

This study provides important insights into the prevalence and distribution of *C.* *sinensis* infection in animal hosts across China. The findings reveal spatio-temporal disparities in the infection rates, with a significant decline observed in areas with low human prevalence, while high prevalence persists in regions with higher human infection rates. The concentration of animal infections in the eastern regions of China aligns with the known range of primary vectors, emphasizing the role of environmental factors such as temperature and precipitation in shaping the distribution of the parasite.

This study calls for a concerted effort to implement One Health-based comprehensive measures in high epidemic areas, along with continued monitoring and control efforts, to effectively reduce the burden of *C.* *sinensis* infection in both human and animal populations. By taking a multidisciplinary approach and collaborating across sectors, we can make significant strides toward eradicating clonorchiasis and improving the health of both humans and animals in China.

### Supplementary Information

**Additional file 1: Figure S1.** Forest plots of the prevalence of *Clonorchis* *sinensis* in snails in China

**Additional file 2: Figure S2.** Forest plots of the prevalence of *Clonorchis* *sinensis* in the second intermediate hosts in China

**Additional file 3: Figure S3.** Forest plots of the prevalence of *Clonorchis* *sinensis* in animal reservoirs in China

**Additional file 4: Figure S4a.** Funnel plot for assessing publication bias in studies reporting infection in animal hosts. (a) *Clonorchis* *sinensis* in snails.

**Additional file 5: Figure S4b.** Funnel plot for assessing publication bias in studies reporting infection in animal hosts. (b) *C.* *sinensis* in the second intermediate hosts.

**Additional file 6: Figure S4c.** Funnel plot for assessing publication bias in studies reporting infection in animal hosts. (c) *C.* *sinensis* in animal reservoirs.

**Additional file 7: Figure S5.** Distribution of *Alocinma* *longicornis*, *Bithynia* *fuchslana*, and *Parafossarulus* *striatulus* in China. The diagonal lines in all maps are the Heihe-Tengchong Line (Hu Line)

**Additional file 8: Figure S6.** Environmental characteristics of regions reporting the existence of *Alocinma* *longicornis*, *Bithynia* *fuchslana*, and *Parafossarulus* *striatulus* in China.

**Additional file 9: Table S1.** Human *Clonorchis* *sinensis* infection in national surveys of important human parasitic diseases in China.

**Additional file 10: Table S2.** Publications reporting *Clonorchis* *sinensis* infection in the first intermediate hosts in China.

**Additional file 11: Table S3.** Publications reporting *Clonorchis* *sinensis* infection in the second intermediate hosts in China.

**Additional file 12: Table S4.** Publications reporting *Clonorchis* *sinensis* infection in animal reservoirs in China.

**Additional file 13: Table S5.** Multivariable meta-regression analysis for *Clonorchis* *sinensis* infection in the first intermediate hosts, the second intermediate hosts, and animal reservoirs.

**Additional file 14: Table S6.** Sensitivity analysis of the pooled prevalence of *Clonorchis* *sinensis* in animal hosts.

**Additional file 15: Table S7.** Sensitive analysis for spatio-temporal disparity of *Clonorchis* *sinensis* in animals in China.

## Data Availability

The data presented in this study are available on request from the author.

## Abbreviations

PLAD: Province
*CI*: Confidence interval
QM: The regression model heterogeneity
QE: The residual error heterogeneity
*SD*: Standard deviation
Min: Minimum
Max: Maximum
