# Supplementary figures and images for: Spatio-temporal disparities of Clonorchis sinensis infection in animal hosts in China: a systematic review and meta-analysis
Source: Infect Dis Poverty. 2023 Oct 17;12:97. doi: 10.1186/s40249-023-01146-4 (PMC10580589; doi:10.1186/s40249-023-01146-4)

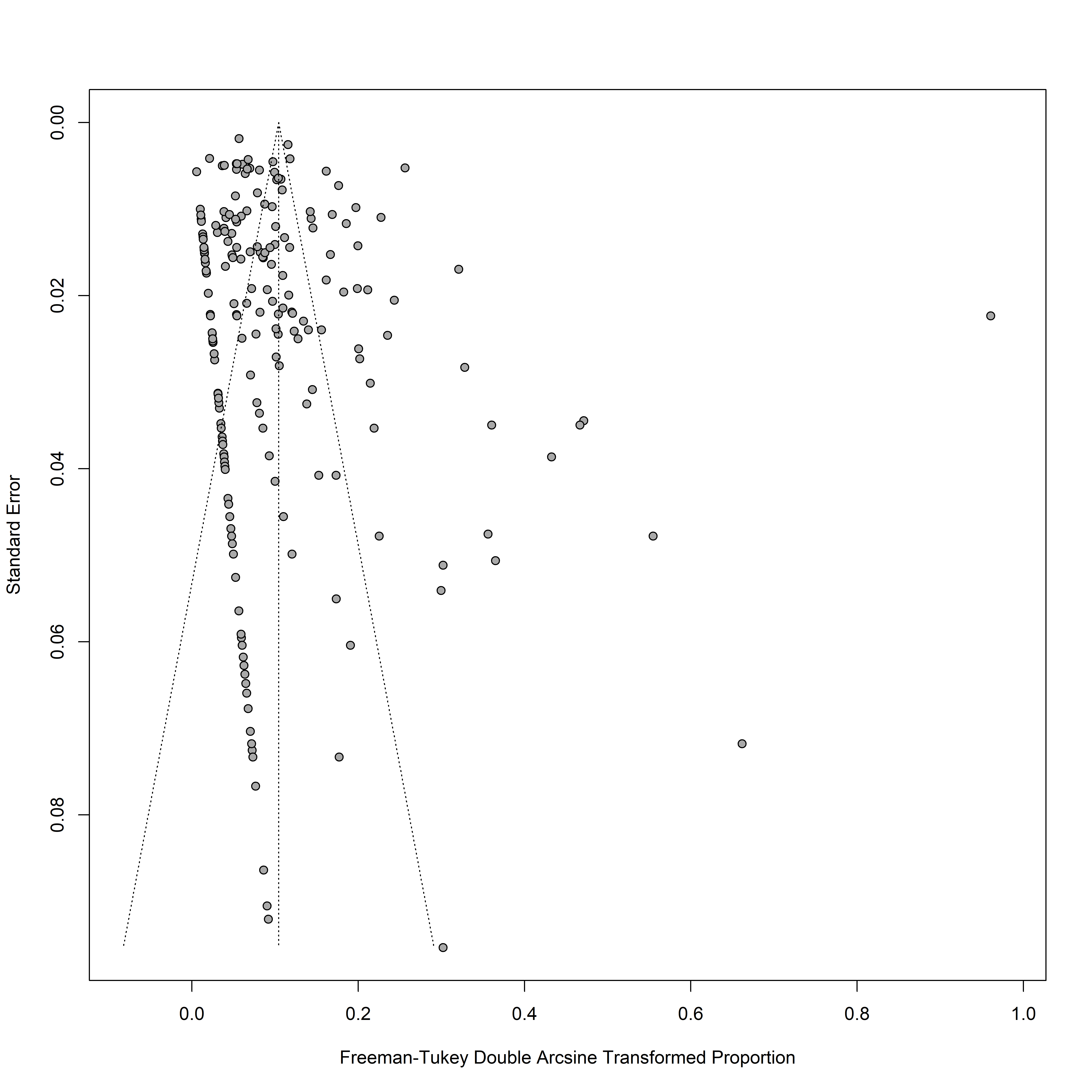

Supplement: Supplementary file 4 — Additional file 4: Figure S4a. Funnel plot for assessing publication bias in studies reporting infection in animal hosts. (a) Clonorchis sinensis in snails. [file 40249_2023_1146_MOESM4_ESM.png]

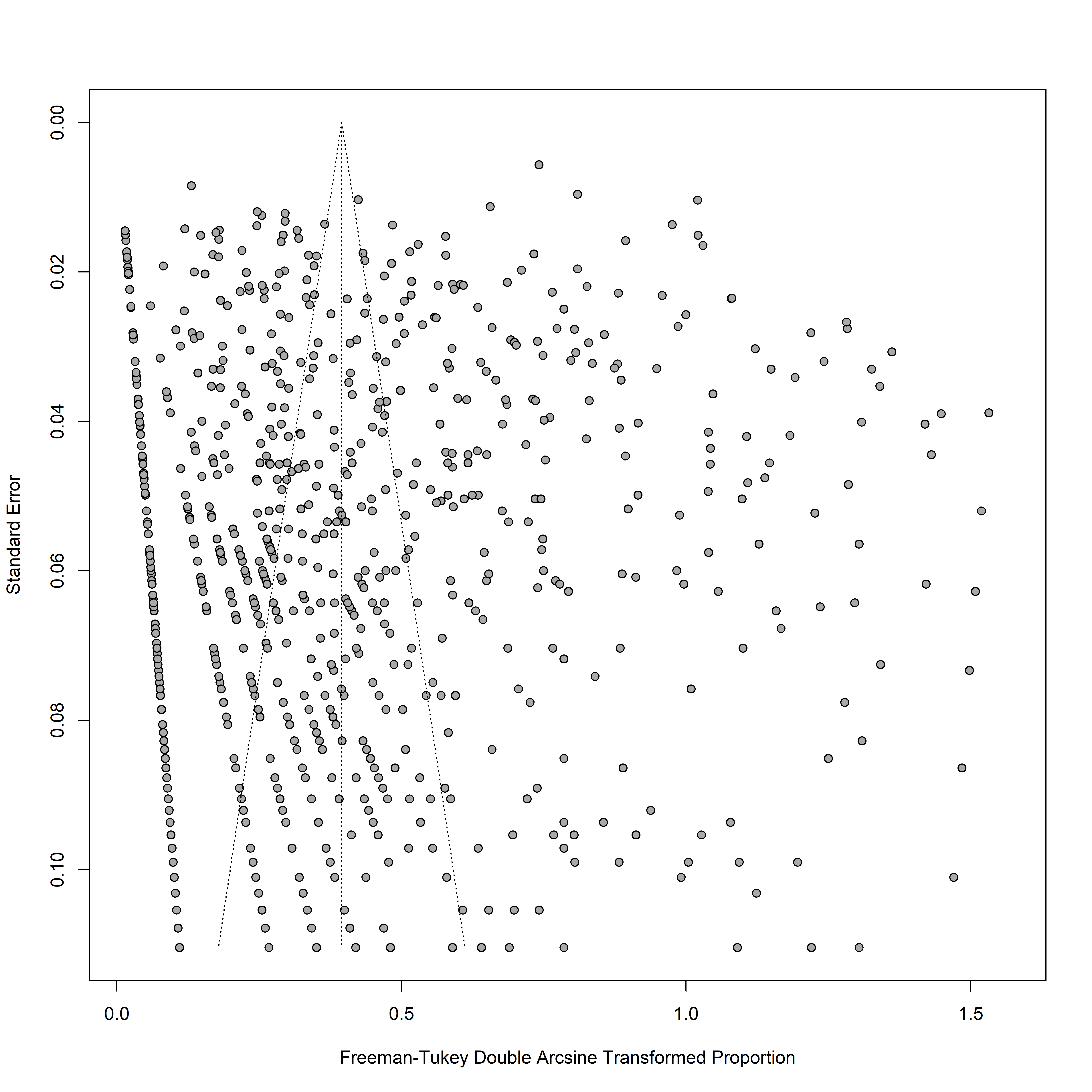

Supplement: Supplementary file 5 — Additional file 5: Figure S4b. Funnel plot for assessing publication bias in studies reporting infection in animal hosts. (b) C. sinensis in the second intermediate hosts. [file 40249_2023_1146_MOESM5_ESM.png]

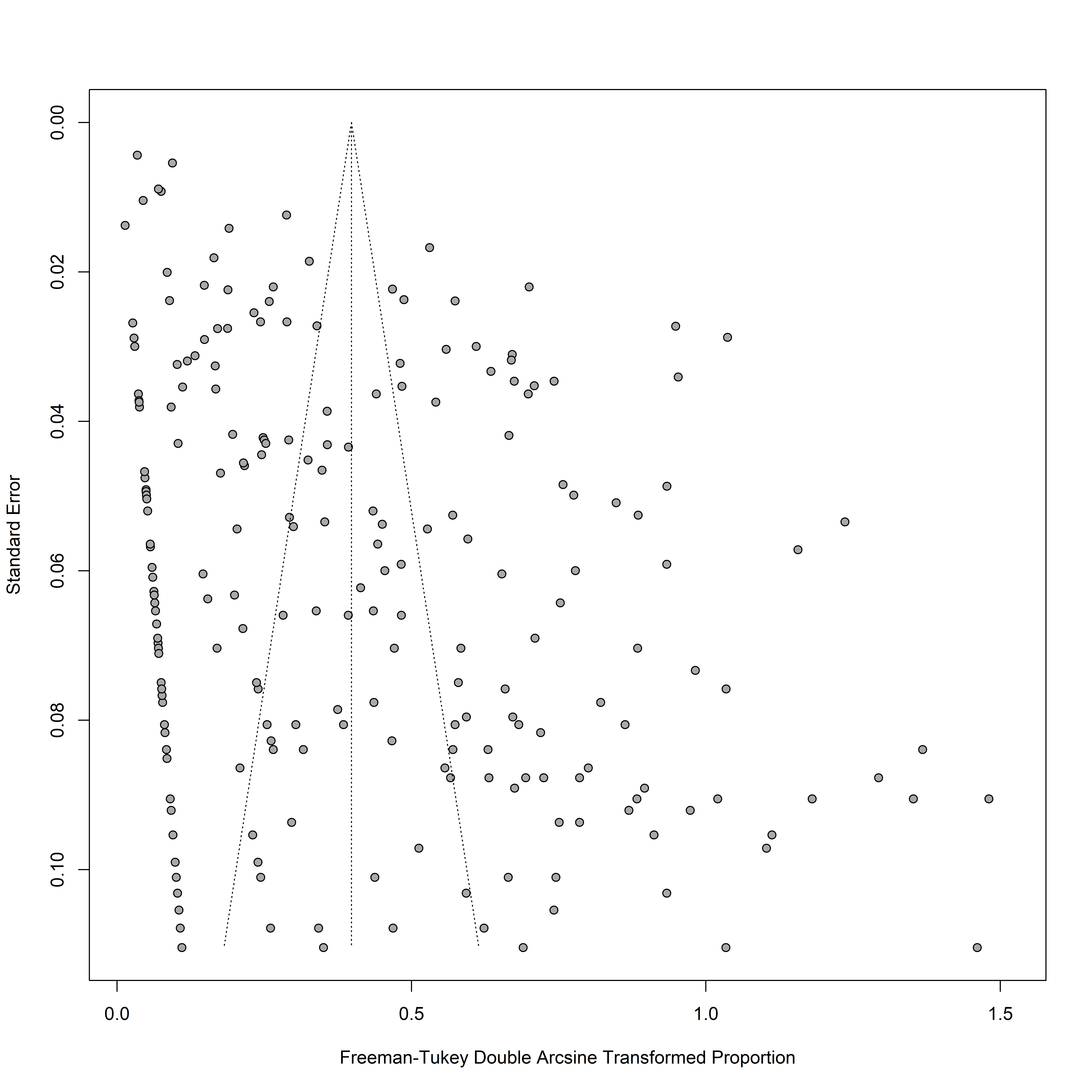

Supplement: Supplementary file 6 — Additional file 6: Figure S4c. Funnel plot for assessing publication bias in studies reporting infection in animal hosts. (c) C. sinensis in animal reservoirs. [file 40249_2023_1146_MOESM6_ESM.png]

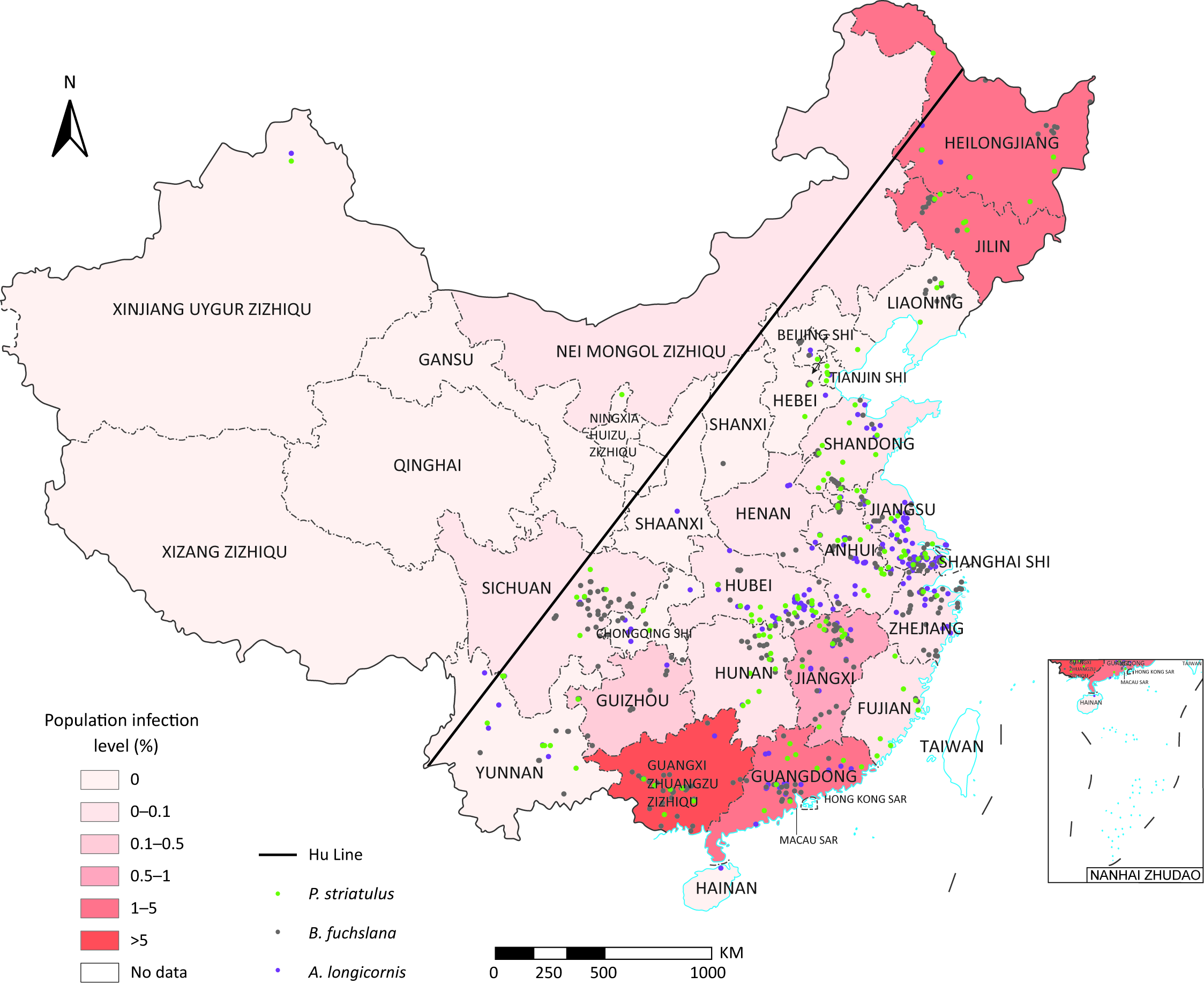

Supplement: Supplementary file 7 — Additional file 7: Figure S5. Distribution of Alocinma longicornis, Bithynia fuchslana, and Parafossarulus striatulus in China. The diagonal lines in all maps are the Heihe-Tengchong Line (Hu Line) [file 40249_2023_1146_MOESM7_ESM.tif]
